# Global, Regional, and National Burden of Breast Cancer Attributable to Low Physical Activity in Women of Reproductive Age: Historical Trends from 1990 to 2021 and Projections to 2035

**DOI:** 10.34172/aim.34998

**Published:** 2025-11-01

**Authors:** Cai Bingliang, Li Jinhao, Liao Haijian, Jiang Yi, He Zhifeng, Yang Chuansheng

**Affiliations:** ^1^Guangdong Medical University, Zhanjiang, China; ^2^Department of Breast, Thyroid and Head-Neck Surgery, Yuebei People’s Hospital Affiliated to Shantou University Medical College, Shaoguan, China; ^3^Yuebei People’s Hospital Joint Postgraduate Training Base, Guangdong Medical University, Shaoguan, China

**Keywords:** Breast cancer, Global burden of disease, Physical activity, Projections, Women of reproductive age

## Abstract

**Background:**

This study analyzes the global patterns and trends of breast cancer attributable to low physical activity (LPA) among women of reproductive age (1990–2021) using Global Burden of Disease (GBD) data, providing quantitative evidence for prioritizing physical-activity interventions to reduce LPA-attributable breast-cancer burden, specifically among women of reproductive age.

**Methods:**

This study used data from the GBD Study 2021. We analyzed the changes in the burden of LPA-related breast cancer in women of reproductive age at the global, regional, and national levels from 1990 to 2021. The Bayesian age-period-cohort (BAPC) model was used to predict future trends. Decomposition analysis using Das Gupta’s framework explored the factors driving changes in LPA-attributable breast-cancer burden among reproductive-age women.

**Results:**

From 1990 to 2021, in high sociodemographic index (SDI) regions, deaths and disability-adjusted life years (DALYs) of breast cancer in women of reproductive age attributable to LPA showed a decreasing trend, with corresponding estimated annual percentage change (EAPC) values of -1.20 (95% CI: -1.29, -1.10) and -1.05 (95% CI: -1.14, -0.96), respectively. Globally, deaths increased by 68% and DALYs by 69% between 1990 and 2021, while low-SDI countries showed the steepest relative rise in age-standardized mortality (EAPC=1.04, 95 % CI: 0.83–1.25) and DALY rates (EAPC=1.08, 95 % CI: 0.87–1.29). Decomposition analysis indicated that population growth was the main driver of the increasing global breast cancer burden. The BAPC model predicted that from 2022 to 2035, the global burden of breast cancer in women of reproductive age attributable to LPA would continue to rise, with age-standardized mortality rates (ASMRs) projected to increase by 7.1 % (to 0.15 per 100,000) and age-standardized DALY rates (ASDRs) by 4.2 % (to 7.3 per 100,000) by 2035.

**Conclusion:**

Globally, the burden of breast cancer in women of reproductive age attributable to LPA has shown an increasing trend, with significant regional disparities. Our findings underscore the importance of physical activity in controlling the burden of breast cancer among reproductive-age women.

## Introduction

 Breast cancer still ranks among the leading causes of cancer-related deaths among women worldwide. Its incidence among women of reproductive age (defined by the WHO as 15‒49 years) has risen markedly over the past three decades. Between 1990 and 2021, the global burden of breast cancer among reproductive-age women has continued to escalate, with incident cases rising from 256,715 to 561,438 and deaths increasing from 78,285 to 129,405, imposing parallel burdens on fertility, maternal health and household economies.^1^ Breast cancer patients of reproductive age showed a higher prevalence of germline pathogenic variants, especially in BRCA1/2.^2^ This underscores the importance of early genetic testing and tailored screening, including annual breast MRI from age 25, mammography and ultrasound. Early-onset breast cancers exhibit high grade, triple-negative subtype and Ki-67 above 30%, associated with recurrence, earlier metastasis and poorer overall survival.^3^ A large retrospective study indicated that pregnancy after breast cancer did not adversely affect disease-free survival, provided that conception occurs at least 12–24 months after treatment completion.^4^ Consequently, international guidelines mandate early fertility counseling prior to therapy initiation, underscoring the need for tailored management in this age group.^5,6^

 Of particular concern is the established association between low physical activity (LPA) and breast cancer risk in reproductive-age women, which has become a focal point of contemporary epidemiological research. Substantial evidence indicates that LPA is a modifiable risk factor in multiple non-communicable diseases, including diabetes, colon cancer, and notably, breast cancer.^7,8^ Epidemiological studies have demonstrated that LPA not only correlates significantly with breast cancer incidence but also adversely affects patients’ quality of life and prognosis.^9,10^ Furthermore, compelling research and Global Burden of Disease (GBD) study has linked LPA to increased all-cause mortality and breast cancer-specific mortality among patients.^11,12^ However, the current GBD study covers all ages only up to 2019 without BAPC projections, and population-based estimates of the LPA-attributable breast-cancer burden specifically for women of reproductive age remain lacking, leaving the magnitude and geographic distribution of this preventable risk over time still uncertain.

 To fill this void, we leveraged GBD 2021 data to quantify the global, age-specific burden from 1990 to 2021 and projected it to 2035, providing the evidence base required for targeted physical-activity interventions. This research aims to provide policymakers and public health researchers with actionable insight to guide optimal allocation of physical activity resources and inform strategic initiatives for breast cancer prevention and control.

## Materials and Methods

###  Data Collection

 All data for this study were obtained from the GBD Study 2021conducted by the Institute for Health Metrics and Evaluation (IHME) at the University of Washington. The data were obtained by means of the Global Health Data Exchange (GHDx) platform (https://vizhub.healthdata.org/gbd-results/). This study was a secondary analysis of publicly available, population-level data, applying the comparative risk assessment (CRA) framework to estimate the burden of breast cancer attributable to LPA among women of reproductive age (15–49 years) from 1990 to 2021.

 We extracted burden metrics (death counts and disability-adjusted life years, DALYs) for breast cancer in women of reproductive age (15‒49 years) attributable to LPA from 1990 to 2021. Age-standardized rates (ASRs) for mortality and DALYs were computed per 100,000 population using the GBD criteria for the population. Uncertainty intervals (UIs) were obtained through 1,000 Monte-Carlo simulations. Additionally, 204 countries and territories were categorized into 21 GBD regions and sociodemographic index (SDI) groups.^13^ The SDI, a composite measure of income, education, and fertility, ranges from 0 to 1 and is calculated as the geometric mean of the total fertility rate under 25, mean years of schooling for those aged ≥ 15 years, and lag-distributed income per capita. Based on the 2021 GBD ranking, the 204 countries and territories were divided into five quintiles: low [0–0.466), low-middle [0.466–0.619), middle [0.619–0.712), high-middle [0.712–0.810), and high [0.810–1.000) SDI.^14,15^ For GBD studies, the Institutional Review Board of the University of Washington reviewed and approved a waiver of informed consent (https://www.healthdata.org/research-analysis/gbd).

###  Data Analysis

 The estimated annual percentage change (EAPC) and its 95% confidence intervals (CIs) were used to quantify the burden and temporal trends in ASRs (upward trends are associated with positive values, while downward trends correspond to negative values).^16^ We generated color-coded world maps to depict the 2021 global distribution of age-standardized mortality and DALYs rates (per 100,000 population), while also illustrating the EAPC trends from 1990 to 2021.

 An analysis of temporal trends from 1990 to 2021, for age-standardized DALYs and death counts at the national, regional, and global levels, was performed using linear regression models. The study further stratified the SDI into 21 GBD regions and 204 countries, conducting correlation analyses between age-standardized mortality rates (ASMRs), age-standardized DALY rates (ASDRs), and SDI for LPA-attributable breast cancer in reproductive-age women. Scatter plots for each region depicted temporal changes from 1990 to 2021 (left to right).

 The Joinpoint regression model was employed to analyze trend transitions in rates over time. By identifying Joinpoint locations and segmenting long-term trends, the annual percentage change (APC) was calculated to evaluate shifts in disease burden.

 Evidence suggests that the Bayesian age-period-cohort (BAPC) model provides more accurate predictions of disease burden.^17^ Future incidence and mortality rates for 2022–2035 were forecast with the BAPC model based on GBD 2021 estimates from 1990–2021 (ages 25–49 years). The model applied conventional priors for age, period and cohort effects, and was validated by posterior predictive checks against observed rates in 2015–2021. Uncertainty in the projections was conveyed by median estimates and 95% UIs computed from the 2.5th and 97.5th percentiles of 1,000 posterior simulations, reflecting the full variability in future disease burden.^18^ The model was run in R 4.2.2 using the ‘BAPC’ package.

 All statistical analyses and mappings were performed using the R software (version 4.2.2). A two-sided approach was adopted for all tests, considering *P* < 0.05 as statistically significant.

###  Disease Definition and Exposure Assessment

 Breast cancer cases were defined using ICD-10 codes (C50, D05, etc) and ICD-9 codes (174, 217, etc).^12^ To assess the global health impact of LPA, the GBD 2021 study incorporated data from the Global Physical Activity Questionnaire, International Physical Activity Questionnaire, and other sources.

 Under the CRA framework, LPA was explicitly defined as physical activity below the exposure threshold of < 3,000 MET-min/week (MET = metabolic equivalent of task, representing resting energy expenditure, specifically the oxygen consumption at seated rest, approximately 3.5 mL O_2_/kg/min or 1 kcal/kg/h).^19^ This threshold represents the theoretical minimum risk exposure level used as the reference level for estimating the attributable burden of breast cancer due to physical inactivity. Population attributable fractions were computed and multiplied by the corresponding DALYs to quantify the attributable burden. This metric expressed the proportion of total disease burden that was ascribed to the specific risk factor or combination of risk factors.

###  Decomposition Analysis

 Das Gupta’s decomposition method was applied to quantify how much of the 1990–2021 change in deaths and DALYs from LPA-attributable breast cancer among women aged 15–49 years was attributable to population growth, population aging, and epidemiological change. This approach allows the overall burden variation to be decomposed into these key factors, thereby more clearly revealing how demographic and epidemiological shifts shape trends over time.^15^ Unlike traditional regression techniques that merely describe associations, decomposition analysis quantifies the independent contribution of each driver while controlling for the others. By dissecting these trends, we gained a sharper understanding of the key drivers behind the global breast-cancer burden.

## Results

###  Global Trends of Breast Cancer Deaths and ASMR Attributable to LPA in Women of Reproductive Age

**Figure 1:**
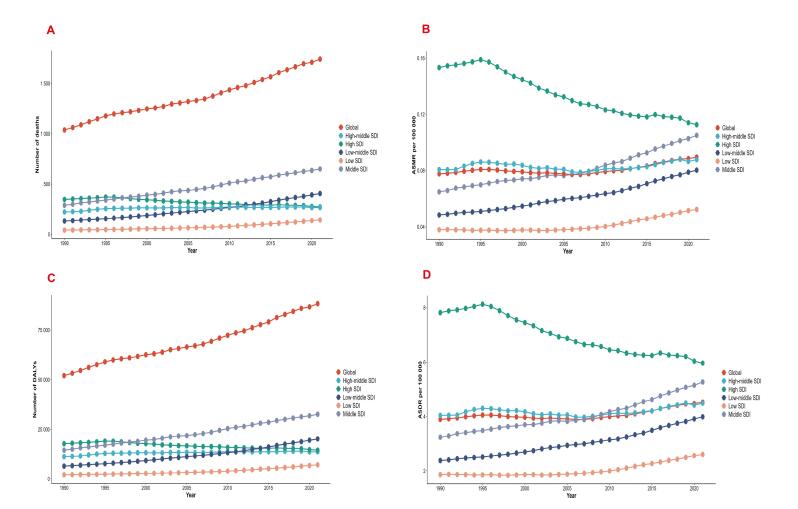
Global deaths **(A)**, ASMR **(B)**, DALYs **(C)** and ASDR **(D)** of Breast Cancer Attributable to Low Physical Activity among Women of Reproductive Age by SDI Region from 1990 to 2021. SDI, Socio-demographic Index; ASMR, Age-standardized mortality rate; DALYs, Disability-adjusted life years; ASDR, Age-standardized DALY rate

**Figure 2:**
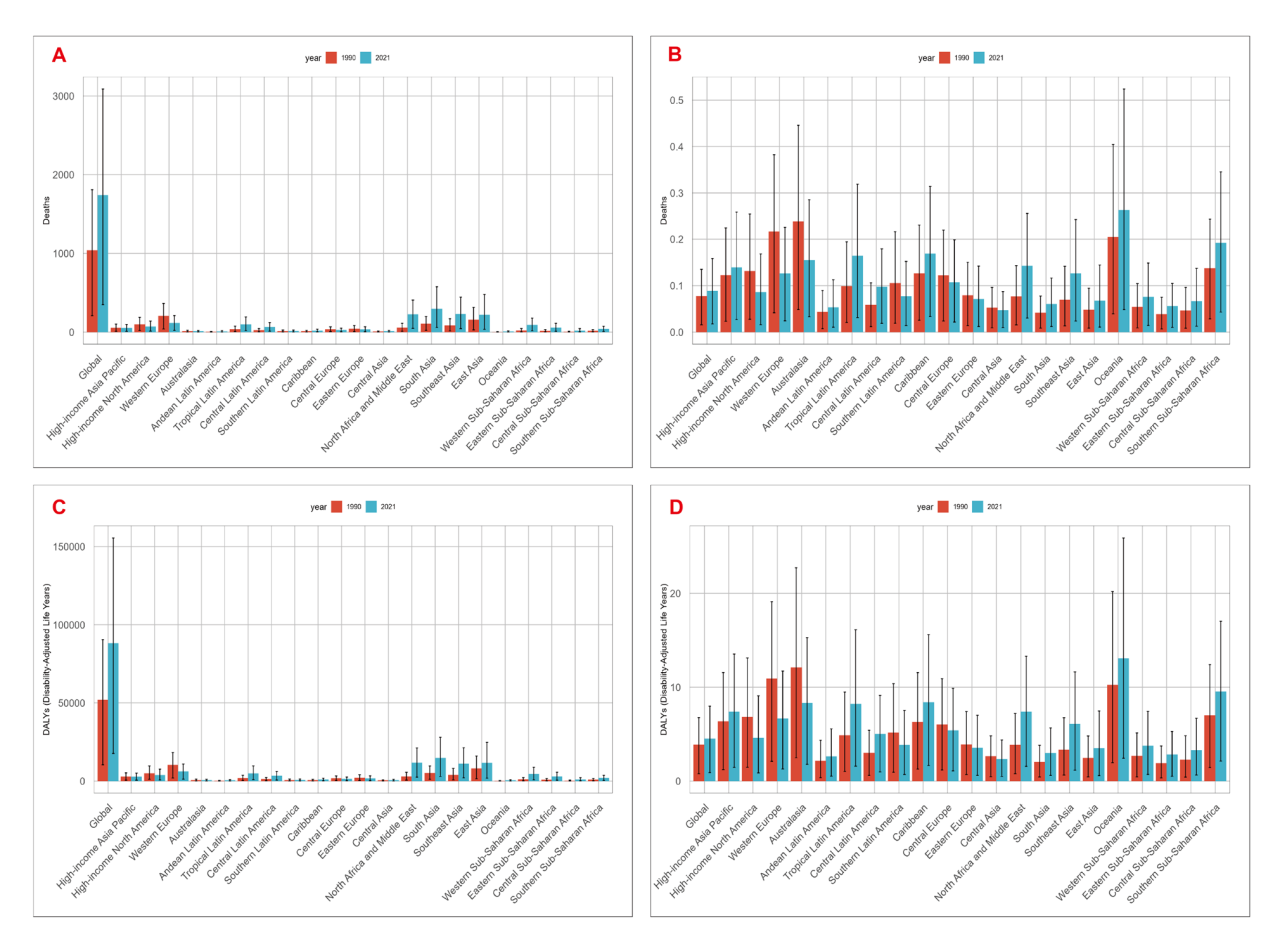
Global Deaths **(A)**, ASMR **(B)**, DALYs **(C)** and ASDR **(D)** of Breast Cancer Attributable to Low Physical Activity among Women of Reproductive Age by GBD Region in 1990 (red) and 2021(blue). Error bars represent 95% uncertainty intervals. ASMR, Age-standardized mortality rate; DALYs, Disability-adjusted life years; ASDR, Age-standardized DALY rate

 At the global level, the number of breast cancer deaths among women of reproductive age increased from 1,038 (95% UI: 210-1,809) in 1990 to 1,743 (95% UI: 348‒3,088) in 2021, representing a 68% increase. The ASMR rose from 0.08 (95% UI: 0.02‒0.14) to 0.09 (95% UI: 0.02‒0.16) per 100,000 population. The global EAPC from 1990 to 2021 was 0.32 (95% CI: 0.21‒0.44) (Table 1, Figure 1A-B, Figure 2A-B).

**Table 1:** Trends in Breast Cancer Deaths at Reproductive Age Associated with Low Physical Activity, 1990‒2021

**Characteristics**	**1990**		**2021**		**1990‒2021**
	**Number of deaths cases (95% UI)**	**Age-standardized death rate/100,000 (95% UI)**	**Number of deaths cases (95% UI)**	**Age-standardized death rate/100,000 (95% UI)**	**EAPC (95% CI)**
Global	1038 (210, 1809)	0.08 (0.02, 0.14)	1743 (348, 3088)	0.09 (0.02, 0.16)	0.32 (0.21, 0.44)
Age					
25‒29 years	25 (5, 48)	0.01 (0.00, 0.02)	39 (8, 73)	0.01 (0.00, 0.03)	0.59 (0.45, 0.73)
30‒34 years	81 (17, 145)	0.04 (0.01, 0.08)	127 (27, 229)	0.04 (0.01, 0.08)	0.03 (-0.14, 0.21)
35‒39 years	182 (35, 320)	0.10 (0.02, 0.18)	272 (52, 493)	0.10 (0.02, 0.18)	-0.28 (-0.47, -0.08)
40‒44 years	308 (61, 543)	0.22 (0.04, 0.39)	495 (101, 923)	0.20 (0.04, 0.37)	-0.44 (-0.62, -0.26)
45‒49 years	441 (91, 793)	0.39 (0.08, 0.70)	809 (160, 1482)	0.34 (0.07, 0.63)	-0.57 (-0.69, -0.45)
SDI region					
High SDI	347 (74, 617)	0.15 (0.03, 0.27)	274 (54, 495)	0.11 (0.02, 0.20)	-1.20 (-1.29, -1.10)
High-middle SDI	224 (44, 387)	0.08 (0.02, 0.14)	268 (50, 499)	0.09 (0.02, 0.16)	0.08 (-0.03, 0.20)
Middle SDI	290 (57, 514)	0.06 (0.01, 0.11)	650 (129, 1156)	0.11 (0.02, 0.19)	1.48 (1.37, 1.58)
Low-middle SDI	132 (28, 240)	0.05 (0.01, 0.09)	406 (86, 734)	0.08 (0.02, 0.15)	1.63 (1.57, 1.70)
Low SDI	42 (8, 80)	0.04 (0.01, 0.07)	144 (29, 257)	0.05 (0.01, 0.09)	1.04 (0.83, 1.25)

EAPC, Estimated annual percentage change; CI, Confidence interval; UI, Uncertainty interval; SDI, Sociodemographic index. The full range of the raw data can be found in Table S1.

###  SDI Regional Trends of Breast Cancer Deaths and ASMR Attributable to LPA in Women of Reproductive Age

 At the SDI regional level in 2021, the majority of LPA-attributable breast cancer deaths among reproductive-age women occurred in middle and low-middle SDI regions. High SDI regions showed declining trends in both death counts and ASMR (EAPC: -1.20, 95% CI: -1.29 to -1.10), while high-middle SDI regions showed no significant changes (EAPC: 0.08, 95% CI: -0.03 to 0.20). In contrast, middle, low-middle and low SDI regions experienced significant increases in both death counts and ASMR, with EAPC values of 1.48 (95% CI: 1.37‒1.58), 1.63 (95% CI: 1.57‒1.70), and 1.04 (95% CI: 0.83‒1.25) respectively. Notably, death counts in low-middle SDI regions surpassed those in high SDI regions by 2010 and continued to show an upward trend (Table 1, Figures 1A-B).

###  Geographic Regional Trends of Breast Cancer Deaths and ASMR Attributable to LPA in Women of Reproductive Age

 At the regional level in 1990, LPA-attributable breast cancer deaths were primarily concentrated in Western Europe (207 deaths) and East Asia (161 deaths), together accounting for over 30% of global deaths. By 2021, South Asia (299 deaths), Southeast Asia (232 deaths), and North Africa and the Middle East (228 deaths) became the top three regions with the highest death burden. From 1990 to 2021, Western Europe (EAPC: -1.80, 95% CI: -1.89 to -1.71), high-income North America (EAPC: -1.42, 95% CI: -1.54 to -1.30), Australasia (EAPC: -1.51, 95% CI: -1.62 to -1.41), Central Asia (EAPC: -0.44, 95% CI: -0.67 to -0.22), Central Europe (EAPC: -0.87, 95% CI: -1.10 to -0.63), Eastern Europe (EAPC: -1.19, 95% CI: -1.52 to -0.86), and Southern Latin America (EAPC: -1.16, 95% CI: -1.36 to -0.95) showed significant declining trends in ASMR. Conversely, the remaining GBD regions exhibited increasing ASMR trends, with North Africa and the Middle East (EAPC: 2.24, 95% CI: 2.11‒2.37), Tropical Latin America (EAPC: 1.48, 95% CI: 1.42‒1.54), and Oceania (EAPC: 0.83, 95% CI: 0.73‒0.92) showing the most substantial increases (Figures 2A-B, details in Table S1).

###  National Trends of Breast Cancer Deaths and ASMR Attributable to LPA in Women of Reproductive Age

 At the national level in 2021, China had the highest number of LPA-attributable breast cancer deaths (205, 95% UI: 32‒445), followed by India (193, 95% UI: 38‒392) and Indonesia (133, 95% UI: 23‒278). The American Samoa had the highest ASMR (0.75 per 100,000, 95% UI: 0.14‒1.42), while Benin consistently had the lowest ASMR (0.01 per 100,000, 95% UI: 0.00-0.03). Among the 204 analyzed countries, 130 showed increasing ASMR trends, with American Samoa showing the most rapid increase (EAPC: 3.16, 95% CI: 2.98‒3.34) and United Kingdom showing the most significant decline (EAPC: -2.42, 95% CI: -2.53 to -2.31) (Table 1, Figures S1A-C).

###  Age-Stratified Trends of Breast Cancer Deaths and ASMR Attributable to LPA in Women of Reproductive Age

**Figure 3:**
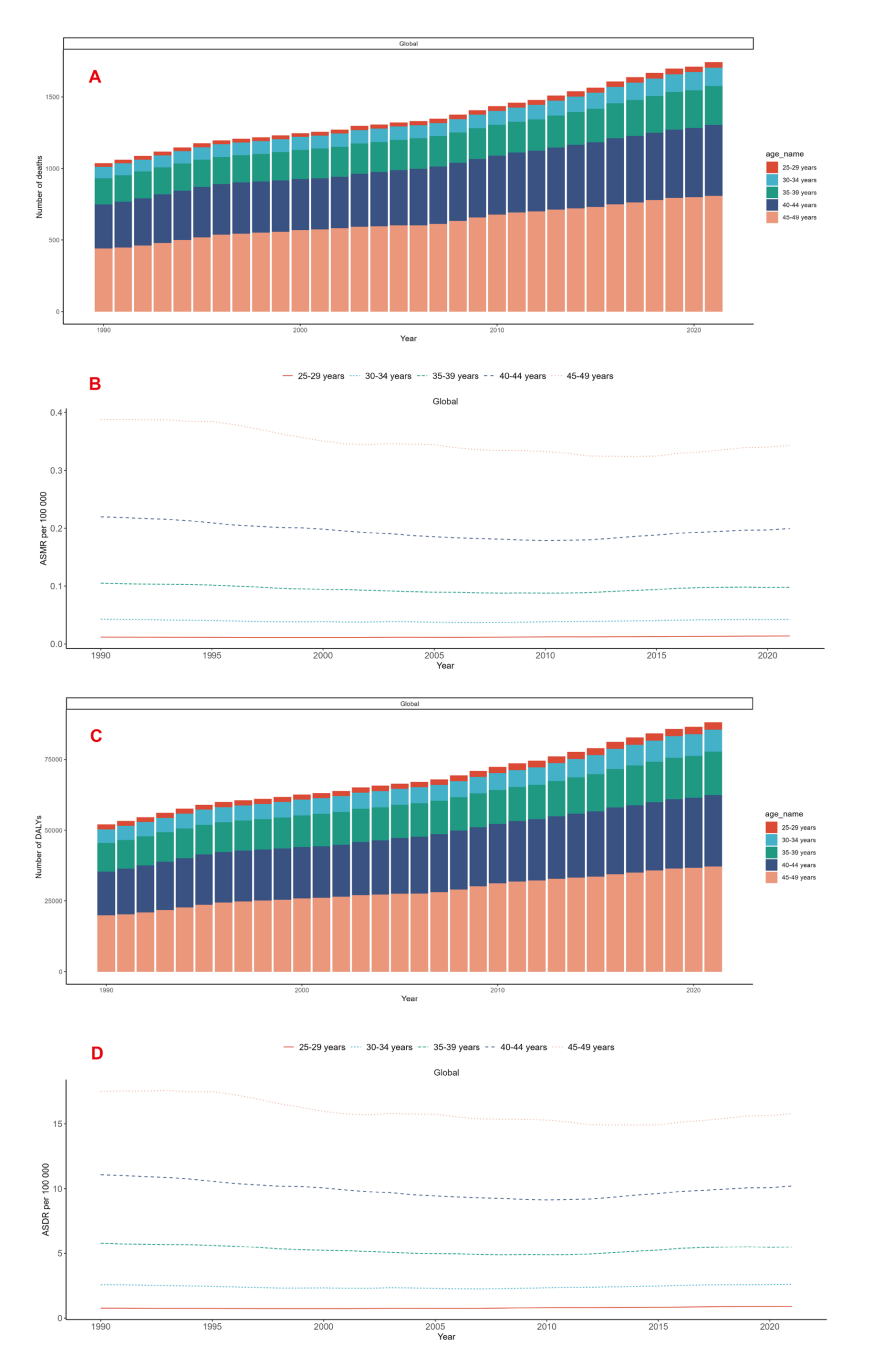
Global Deaths **(A)**, ASMR **(B)**, DALYs **(C)** and ASDR **(D)** of Breast Cancer Attributable to Low Physical Activity among Reproductive-Aged Women Globally in 5 Age Groups from 1990 to 2021. ASMR, Age-standardized mortality rate; DALYs, Disability-adjusted life years; ASDR, Age-standardized DALY rate

 From 1990 to 2021, absolute breast cancer death counts increased across all studied age groups globally. For example, the 25‒29 age group showed a significant upward trend (EAPC: 0.59, 95% CI: 0.45‒0.73), with deaths increasing from 25 to 39 (56% increase). The 45‒49 age group saw deaths more than double (from 441 to 809, 83.5% increase). However, ASMR showed divergent trends: the 25‒29 age group maintained relatively stable ASMR (0.01 per 100,000 in both 1990 and 2021), while older age groups, particularly the 45‒49 group, showed significant ASMR declines (from 0.39 to 0.34 per 100,000, -12.8% change; EAPC: -0.57%, 95% CI: -0.69 to -0.45) (Table 1, Figures 3A-B).

###  Global Trends of Breast Cancer DALYs and ASDR Attributable to LPA in Women of Reproductive Age

 At the global level, breast cancer DALYs increased from 52,042 (95% UI: 10,459‒90,559) in 1990 to 88,198 (95% UI: 17,665‒155,543) in 2021, representing a 69.5% increase, while the ASDR rose from 3.89 (95% UI: 0.78‒6.77) to 4.53 (95% UI: 0.91‒7.98) per 100,000 population. The EAPC was 0.36 (95% CI: 0.24‒0.47) (Table 2, Figures 1C-D, Figures 2C-D).

**Table 2:** DALYs Trends for Breast Cancer at Reproductive Age Associated with Low Physical Activity, 1990‒2021

**Characteristics**	**1990**		**2021**		**1990‒2021**
	**Number of DALYs cases (95% UI)**	**Age-standardized DALYs rate/100000 (95% UI)**	**Number of DALYs Cases (95% UI)**	**Age-standardized DALYs rate/100000 (95% UI)**	**EAPC (95% CI)**
Global	52042 (10459, 90559)	3.89 (0.78, 6.77)	88198 (17665, 155543)	4.53 (0.91, 7.98)	0.36 (0.24, 0.47)
Age					
25‒29 years	1677 (334, 3130)	0.76 (0.15, 1.42)	2637 (503, 4935)	0.91 (0.17, 1.70)	0.65 (0.51, 0.79)
30‒34 years	4891 (1003, 8748)	2.57 (0.53, 4.60)	7792 (1698, 14163)	2.61 (0.57, 4.74)	0.09 (-0.08, 0.27)
35‒39 years	10043 (1942, 17656)	5.79 (1.12, 10.18)	15240 (2898, 27429)	5.49 (1.04, 9.87)	-0.23 (-0.42, -0.03)
40‒44 years	15519 (3091, 27397)	11.07 (2.20, 19.54)	25330 (5202, 47190)	10.21 (2.10, 19.02)	-0.38 (-0.56, -0.21)
45‒49 years	19912 (4119, 35622)	17.50 (3.62, 31.30)	37199 (7285, 68110)	15.79 (3.09, 28.90)	-0.51 (-0.62, -0.40)
SDI region					
High SDI	17722 (3777, 31429)	7.82 (1.67, 13.86)	14506 (2831, 25929)	5.97 (1.16, 10.66)	-1.05 (-1.14, -0.96)
High-middle SDI	11215 (2202, 19421)	4.04 (0.79, 6.99)	13689 (2604, 25135)	4.49 (0.85, 8.24)	0.17 (0.05, 0.28)
Middle SDI	14462 (2822, 25829)	3.23 (0.63, 5.78)	32589 (6509, 58593)	5.27 (1.05, 9.47)	1.49 (1.39, 1.59)
Low-middle SDI	6492 (1359, 11800)	2.38 (0.50, 4.32)	20172 (4242, 36273)	3.98 (0.84, 7.16)	1.67 (1.59, 1.74)
Low SDI	2074 (412, 3918)	1.86 (0.37, 3.51)	7131 (1448, 12797)	2.60 (0.53, 4.67)	1.08 (0.87, 1.29)

DALYs, Disability-Adjusted Life Years; EAPC, Estimated annual percentage change; CI, Confidence interval; UI, Uncertainty interval; SDI, Socio- demographic index. The full range of the raw data can be found in Table S2.

###  SDI Regional Trends of Breast Cancer DALYs and ASDR Attributable to LPA in Women of Reproductive Age

 At the SDI regional level from 1990 to 2021, high SDI regions showed significant declines in both LPA-attributable breast cancer DALYs (from 17,722 to 14,506) and ASDR (from 7.82 to 5.97), with an EAPC of -1.05 (95% CI: -1.14 to -0.96). In contrast, high-middle, middle, low-middle and low SDI regions all demonstrated increasing trends in both DALYs and ASDR. Notably, middle SDI regions exhibited the most rapid ASDR increase (from 3.23 to 5.27; EAPC = 1.49; 95% CI: 1.39‒1.59), followed by low-middle SDI regions (from 2.38 to 3.98; EAPC = 1.67; 95% CI: 1.59‒1.74). Low SDI regions maintained the lowest ASDR levels but showed gradual upward trends (EAPC = 1.08; 95% CI: 0.87‒1.29). High-middle SDI regions demonstrated stable DALYs but moderate ASDR growth (EAPC = 0.17; 95% CI: 0.05‒0.28) (Table 2, Figures 1C-D).

###  Geographic Regional Trends of Breast Cancer DALYs and ASDR Attributable to LPA in Women of Reproductive Age

 At the regional level in 1990, Western Europe and East Asia had the highest LPA-attributable breast cancer DALYs (10,424 and 8,216 respectively), while by 2021, the highest burden regions were South Asia, North Africa and Middle East, and East Asia (14,713, 11,804 and 11,607 respectively). In 1990, Australasia had the highest ASDR (12.12 per 100,000; 95% UI: 2.49‒22.72), which shifted to Oceania by 2021 (13.07 per 100,000; 95% UI: 2.43‒25.90). High-income North America, Western Europe, Australasia, Eastern Europe, Central Europe, Central Asia and Southern Latin America showed declining ASDR trends, with EAPC ranging from -1.63 to -0.50; whereas Southeast Asia, Oceania, Caribbean, Andean Latin America, Central Latin America, North Africa and Middle East, Tropical Latin America, South Asia and Southern Sub-Saharan Africa regions demonstrated increasing ASDR trends, with EAPC ranging from 0.39 to 2.32 (Figure 2C-D, details in Table S2).

###  National Trends of Breast Cancer DALYs and ASDR Attributable to LPA in Women of Reproductive Age

 At the national level in 2021, India (9511), China (10692) and Indonesia (6299) bore the heaviest global breast cancer DALYs burden. In 1990, the United Kingdom (20.54 per 100,000) and Barbados (19.39 per 100,000) had the highest ASDR, which shifted to Marshall Islands (31.48 per 100,000) and American Samoa (36.76 per 100,000) by 2021. EAPC trends showed marked variation: significant declines in many European countries (e.g. Denmark: -3.43; Germany: -1.72; UK: -2.25), stable or slightly decreasing trends in Romania (-0.12) and Georgia (-0.52), versus substantial increases across Asian (e.g. Thailand: 2.59; Philippines: 2.01; Bangladesh: 3.03; Turkey: 6.29), African (e.g. Libya: 3.58; Kenya: 2.65) and American countries (e.g., Jamaica: 2.85; Colombia: 2.21; Belize: 2.46) (Table 2, Figures S2A-C).

###  Age-Stratified Trends of Breast Cancer DALYs and ASDR Attributable to LPA in Women of Reproductive Age

 From 1990 to 2021, absolute breast cancer DALYs increased across all studied age groups globally. For example, the 30‒34 age group saw DALYs rise from 4891 to 7792, while the 45‒49 age group increased from 19,912 to 37,199. However, ASDR showed divergent trends: the 30‒34 age group maintained nearly stable ASDR (from 2.57 to 2.61 per 100,000), while older age groups demonstrated significant declines, particularly the 45‒49 age group (from 17.50 to 15.79 per 100,000, change rate: -9.8%; EAPC: -0.51%, 95% CI: -0.62 to -0.40). Conversely, the 25‒29 age group showed significant ASDR increases (from 0.76 to 0.91 per 100,000; EAPC: 0.65, 95% CI: 0.51‒0.79) (Table 2, Figures 3C-D).

###  Correlation Analysis Between Death rates/ DALYs rates of Breast Cancer in Women of Reproductive Age and SDI ​​

 Further analysis aimed to explore the correlation between the burden of breast cancer attributable to LPA in women of reproductive age across 21 GBD regions and the Socio-demographic Index (SDI) in GBD 2021. The results demonstrated significant positive correlations between SDI and both ASMR (r = 0.5056, *P* < 0.001) and ASDR (r = 0.5212, *P* < 0.001).

**Figure 4:**
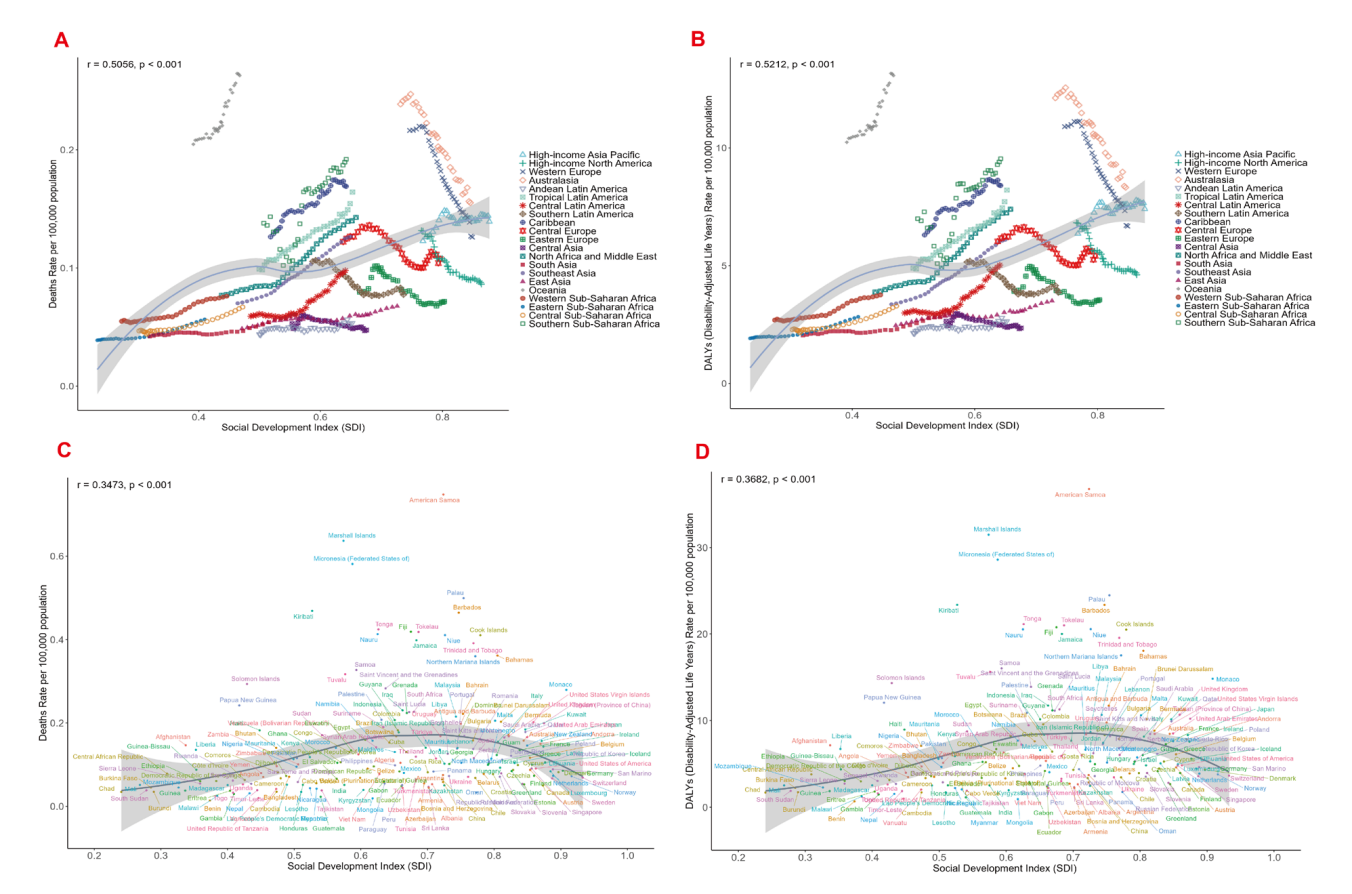
Association between Breast Cancer Burden and SDI in Women of Reproductive Age Attributable to Low Physical Activity in Different Regions and Countries. **(A)** Scatter plot of association between death rate and SDI in breast cancer in 21 GBD regions from 1990 to 2021. **(B)** Scatter plot of association between DALY rate and SDI in breast cancer in 21 GBD regions from 1990 to 2021. **(C) **Scatter Plot of Breast Cancer Death Rates Correlated with SDI Across 204 Countries or Regions in 2021. **(D)** Scatter Plot of Breast Cancer DALY Rates Correlated with SDI Across 204 Countries or Regions in 2021. DALY, Disability-adjusted life-years; GBD, Global Burden of Diseases, Injuries, and Risk Factors Study. SDI, Socio-demographic index

 For death rates:

- In high-SDI regions such as Western Europe and Australasia, death rates were substantially higher than predicted by the SDI correlation, though the trend gradually approached the expected values with increasing SDI.
- In low-SDI regions (e.g. Southern Sub-Saharan Africa and Eastern Sub-Saharan Africa), death counts were markedly lower than predicted by SDI-based relationships (Figure 4A).
- For DALYs rates:
- Regions like Oceania and Western Europe exhibited values far exceeding SDI-correlated predictions;
- While East Asia and South Asia showed significantly lower DALYs rates than expected (Figure 4B).

 A similar analysis across 204 countries and territories in 2021 revealed significant positive correlations between SDI and both death rates/DALYs rates of LPA-attributable breast cancer in reproductive-age women (both *P* < 0.001). Death rates and DALYs rates burdens showed linear increases with rising SDI (ASMR: r = 0.3473; ASDR: r = 0.3682), but this trend plateaued and began declining in regions with SDI > 0.7. High-SDI countries (e.g. Barbados, Palau, American Samoa) exhibited death rates/DALYs rates substantially above the predicted trends, whereas low-middle-SDI nations (e.g. Burundi, Burkina Faso, Togo) showed lower-than-expected rates (Figures 4C-D).

 In summary, these findings confirm a strong positive correlation between SDI and the burden (death rates/DALYs rates) of LPA-attributable breast cancer in reproductive-age women globally. Despite statistically significant linear trends with SDI elevation, substantial heterogeneity persists across nations and regions, as evidenced by wide data dispersion in both figures.

###  Joinpoint Regression Analysis of Mortality and DALY Rate Trends in Reproductive-Age Breast Cancer Patients with Low Physical Activity (1990‒2021)

**Figure 5:**
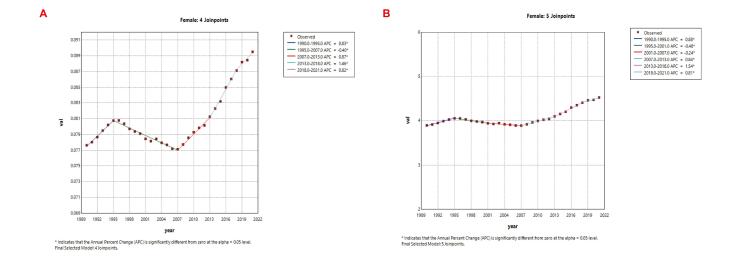
Temporal Trends in Breast Cancer Burden Attributable to Low Physical Activity among Women of Reproductive Age from 1990 to 2021. **(A)** ASMR of breast cancer attributable to low physical activity among women of reproductive age from 1990 to 2021. **(B) **ASDR of breast cancer attributable to low physical activity among women of reproductive age from 1990 to 2021. ASMR, Age-standardized mortality rate; ASDR, Age-standardized DALY rate; APC, Annual percent change

 Joinpoint regression analysis was applied to examine trends in mortality and DALY rates among reproductive-age women with breast cancer attributable to LPA from 1990 to 2021. Analysis of the data revealed that death counts showed an overall increasing trend, with a decline observed in 1995, followed by a resurgence in 2007, reaching peak growth between 2013 and 2018. Similarly, DALY rates demonstrated a gradual upward trend, paralleling mortality patterns with declines from 1995 to 2007 and peak growth during 2013‒2018 (Figures 5A-B). These findings indicate that the impact of LPA on breast cancer in reproductive-age women continues to intensify, potentially associated with urbanization, occupational sedentariness, and increased reliance on electronic devices, while exposing insufficient public health system responses. Although the growth rate slowed after 2018, no substantial decline has been observed.

###  Decomposition Analysis

**Figure 6:**
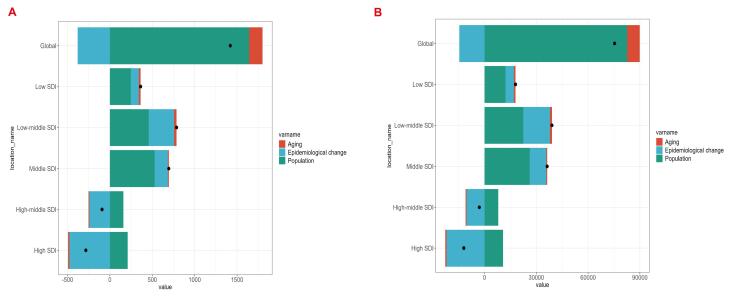
Decomposition Analysis of Deaths** (A) **and DALYs **(B)** in Women of Childbearing Age with Breast Cancer Globally and in five SDI Regions. Black dots indicate the combined effect of these three factors: positive values indicate an increase and negative values indicate a decrease in the corresponding indicator

 In this study, we conducted decomposition analyses at global and five SDI regional levels to assess deaths and DALYs attributable to LPA-associated breast cancer in women of reproductive age. Black dots represent the combined effects of these factors: positive values indicate increases, while negative values denote decreases in corresponding metrics.^20^ The decomposition revealed that population growth and epidemiological changes were the primary drivers of the changing burden. For deaths, population growth accounted for approximately 116% of the net global increase, epidemiological change for about -27%, and aging for roughly 11%; for DALYs, the corresponding contributions were about 110%, -19% and 10%. In low, low-middle and middle SDI settings, population growth remained the dominant contributor, responsible for about three-quarters of the rise, at approximately 68%, 58% and 76% for deaths and 68%, 57% and 72% for DALYs, with epidemiological changes adding a further moderate share of about 26%, 38% and 23% for deaths and 27%, 40% and 26% for DALYs. Conversely, in high-middle and high SDI regions, net reductions occurred because epidemiological improvements outweighed population-driven increases, contributing more than 160% of the net change for both metrics, while aging contributed less than 6% across all quintiles, as detailed in Tables S3 and S4 and shown in Figures 6A-B. Aging demonstrated minimal contributions across all regions, exerting negligible direct impacts. Public health priorities should address population growth and healthcare deficiencies in low SDI regions, while high SDI areas require sustained epidemiological interventions. Globally, dual strategies controlling rapid population growth and optimizing medical resource allocation are essential to mitigate burden disparities across development tiers.

###  Projected Global Burden of Breast Cancer in Women of Reproductive Age by 2035

**Figure 7:**
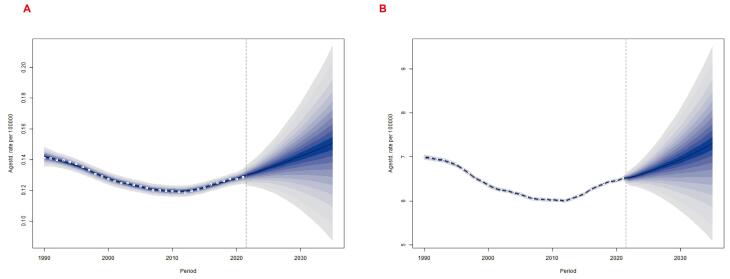
To Predict the Trend of ASMR **(A)** and ASDR **(B)** in Breast Cancer of Reproductive Age Attributed to Low Physical Activity from 2022 to 2035 Based on Bayesian Age-Period-Cohort Model. ASMR, Age-standardized mortality rate; ASDR, Age-standardized DALY rate

 Using the BAPC model, we predicted the global burden of breast cancer attributable to LPA among women of reproductive age from 2022 to 2035. Both ASMR and ASDR were projected to show sustained upward trends over the next decade. The ASMR was expected to increase from approximately 0.13 (95% UI 0.12‒0.14) per 100,000 in 2022 to 0.15 (95% UI 0.09‒0.21) per 100,000 in 2035, representing a growth of about 7.1% in the ASR. Similarly, the ASDR was projected to rise from around 7.0 (95% UI 0.12‒0.14) per 100,000 in 2022 to 7.3 (95% UI 5.10‒9.51) per 100,000 in 2035, reflecting a 4.2% increase in the ASR (Figures 7A-B). These projections indicated that the global burden of breast cancer attributable to LPA among reproductive-age women will continue to worsen over the next decade, posing significant challenges for disease prevention and control. In this context, the development of strategic public health interventions and expanded access to early screening and standardized treatment will be critical to mitigating this escalating disease burden.

## Discussion

 This study estimated the spatiotemporal trends of LPA-associated breast cancer deaths and DALYs at global, regional, and national levels, demonstrating the significant impact of LPA on breast cancer burden among women of reproductive age. Globally, from 1990 to 2021, deaths, DALYs, and corresponding ASMR and ASDR of LPA-associated breast cancer in reproductive-age women all showed increasing trends. This upward trajectory was driven chiefly by population growth and epidemiological change in low- and middle-SDI settings, while high-SDI countries experienced net reductions as documented in our decomposition analysis. Thus, the global rise co-exists with declining burdens in the most developed regions. Because population growth had driven most of the burden increase in low- and middle-income settings, our BAPC global projections likewise anticipated continued rises through 2035 unless these demographic or epidemiological trends were reversed. Our analysis of global, regional, and national trends revealed significant disparities across countries, regions, and socioeconomic strata, underscoring the need for comprehensive strategies addressing demographic and epidemiological factors to counter the growing burden of LPA-attributable breast cancer.

 Our study identified marked variations in LPA-associated burden across SDI regions. In 2021, middle and low-middle SDI regions exhibited higher and rising burden rates, particularly in populous countries like India and China, where uneven resource distribution exacerbates the challenges. Conversely, high-SDI regions showed declining trends. This divergence may stem from higher socioeconomic groups engaging more in leisure-time physical activity (LTPA), while resource-constrained populations face barriers to LTPA and other physical activities.^21,22^ Although high-SDI regions grapple with sedentary lifestyles, their robust healthcare systems were associated with lower attributable mortality, partly reducing the burden that would otherwise be assigned to LPA.^12,23^ However, cross-national inequality analyses highlight disproportionate impacts on low-SDI regions, where healthcare shortages and limited health awareness compound LPA risks alongside comorbidities like malnutrition. Further analyses suggest that LPA’s impact is modulated by socio-environmental factors: high-SDI regions leverage community programs to boost activity levels, whereas low-SDI areas struggle with infrastructure gaps. For instance, Africa’s rising breast cancer incidence correlates with declining activity levels and lifestyle shifts.^24^ Culturally tailored interventions, like Nigeria’s education-based programs linking breast cancer awareness to activity promotion,^24^ demonstrate potential. International collaboration through funding, technology transfer, and training is vital to establish effective activity-promotion mechanisms in low-SDI settings.^25^

 Reproductive-age women, as a high-risk group, exhibit age-specific trends: the 45‒49 cohort peaks in LPA-associated deaths and DALYs, likely due to lifestyle shifts, occupational stress, endocrine changes, and menopausal transitions that reduce activity levels.^24,26^ The LPA-breast cancer link involves multifaceted biological mechanisms. Kim et al found that low-intensity exercise may delay tumorigenesis by inducing apoptosis and suppressing M2 macrophage polarization.^27^ LPA contributes to hormonal imbalance, immune dysfunction, and chronic inflammation, all implicated in breast cancer pathogenesis. Indirectly, LPA elevates risk through BMI and metabolic health, particularly in low- and middle-income countries where high BMI drives DALYs. Evidence confirms inverse correlations between activity levels and breast cancer risk in premenopausal women.^28,29^ Higher physical-activity levels were associated with better body composition and with lower reported recurrence and mortality rates in observational cohorts.^30,31^ Among women of reproductive age, accruing ≤ 2.7 hours per week of moderate-intensity recreational physical activity (equivalent to ≥ 10.75 MET-hours, the threshold defined as “active”) has been associated with a lower breast cancer risk.^32^

 Clinical guideline committees should consider adding a single screening question on weekly moderate-to-vigorous physical activity and advise women who fall below the 3000 MET-min threshold to increase walking or cycling. This one-sentence counselling can be delivered during routine antenatal or primary-care visits at no additional cost and directly aligns with the burden reductions predicted by our model.

## Limitations

 In the GBD study, physical activity data were collected through standardized questionnaires, but self-reported measures are susceptible to social desirability bias, recall errors, and disparities in health literacy which may lead to exposure misclassification and attenuate the true attributable fraction. Second, the CRA assumes universal relative-risk functions across countries and time periods, and GBD outputs provide only overall burden estimates for reproductive-age women, without subtype-specific pathology (e.g. Luminal A/B, HER2 + , triple-negative) or male-specific risk patterns. Third, cancer-registry and vital-statistics coverage remain incomplete in many low- and low-middle-SDI settings, so observed burdens may be underestimated and model inputs subject to geographical bias. Additionally, the Bayesian projections only reflect uncertainty within the model structure and do not capture external disruptions (e.g. new physical-activity policies, breakthrough treatments, or unexpected demographic changes); consequently, the post-2021 estimates should be viewed as scenario-based explorations rather than definitive forecasts. These limitations may collectively bias GBD estimates toward underestimation of the LPA-associated breast-cancer burden, particularly in data-sparse regions.

## Conclusion

 In summary, from 1990 to 2021, while high-SDI regions showed declining trends in LPA-associated breast cancer burden among women of reproductive age, the burden continued to rise globally and in other SDI regions, likely due to inequities in prevention, diagnosis, and treatment resources. Decomposition analysis indicates that population growth is the major driver of increased burden across global and SDI regions, though shifts in epidemiological patterns partially mitigated this trend. Regional and national burdens correlated positively with SDI, with substantial disparities across SDI tiers, reflecting underlying economic disparities, healthcare access gaps, and insufficient policy implementation. Projections based on the BAPC model suggest further global burden escalation by 2035. These findings underscore physical activity’s critical role in mitigating breast cancer burden among reproductive-age women. To address gaps, policymakers should prioritize integrating physical activity promotion into reproductive health services (e.g. personalized guidance during prenatal care) and launching community-based physical activity interventions (e.g. free fitness classes) in low- and middle-income countries.

## Supplementary Files

Supplementary file 1 contains Tables S1-S4 and Figures S1-S2.

## References

[1] Cai Y, Dai F, Ye Y, Qian J (2025). The global burden of breast cancer among women of reproductive age: a comprehensive analysis. Sci Rep.

[2] Dullens B, de Putter R, Lambertini M, Toss A, Han S, Van Nieuwenhuysen E (2020). Cancer surveillance in healthy carriers of germline pathogenic variants in BRCA1/2: a review of secondary prevention guidelines. J Oncol.

[3] Hu X, Myers KS, Oluyemi ET, Philip M, Azizi A, Ambinder EB (2021). Presentation and characteristics of breast cancer in young women under age 40. Breast Cancer Res Treat.

[4] Azim HA Jr, Kroman N, Paesmans M, Gelber S, Rotmensz N, Ameye L (2013). Prognostic impact of pregnancy after breast cancer according to estrogen receptor status: a multicenter retrospective study. J Clin Oncol.

[5] Moragón S, Di Liello R, Bermejo B, Hernando C, Olcina E, Chirivella I (2021). Fertility and breast cancer: a literature review of counseling, preservation options and outcomes. Crit Rev Oncol Hematol.

[6] Lambertini M, Peccatori FA, Demeestere I, Amant F, Wyns C, Stukenborg JB (2020). Fertility preservation and post-treatment pregnancies in post-pubertal cancer patients: ESMO Clinical Practice Guidelines†. Ann Oncol.

[7] Carbone S, Del Buono MG, Ozemek C, Lavie CJ (2019). Obesity, risk of diabetes and role of physical activity, exercise training and cardiorespiratory fitness. Prog Cardiovasc Dis.

[8] Paluska SA, Schwenk TL (2000). Physical activity and mental health: current concepts. Sports Med.

[9] Johnsson A, Broberg P, Johnsson A, Tornberg Å, Olsson H (2015). Physical inactivity increases the risk of endometrial cancer and premenopausal breast cancer. Cancer Res.

[10] Godinho-Mota JCM, Gonçalves LV, Soares LR, Mota JF, Martins KA, Freitas-Junior I (2018). Abdominal adiposity and physical inactivity are positively associated with breast cancer: a case-control study. Biomed Res Int.

[11] Nelson SH, Marinac CR, Patterson RE, Nechuta SJ, Flatt SW, Caan BJ (2016). Impact of very low physical activity, BMI, and comorbidities on mortality among breast cancer survivors. Breast Cancer Res Treat.

[12] Yin X, Zhang T, Zhang Y, Man J, Yang X, Lu M (2022). The global, regional, and national disease burden of breast cancer attributable to low physical activity from 1990 to 2019: an analysis of the Global Burden of Disease Study 2019. Int J Behav Nutr Phys Act.

[13] Zhang R, Fan S, Zhu C, Chen S, Tian F, Huang P (2025). Global trends and patterns in cardiovascular disease burden attributable to low physical activity: a systematic analysis for Global Burden of Disease Study from 1990 to 2021. PLoS One.

[14] Rong J, Cheng P, Li D, Wang X, Zhao D (2024). Global, regional, and national temporal trends in prevalence for depressive disorders in older adults, 1990-2019: An age-period-cohort analysis based on the Global Burden of Disease Study 2019. Ageing Res Rev.

[15] Bai Z, Han J, An J, Wang H, Du X, Yang Z (2024). The global, regional, and national patterns of change in the burden of congenital birth defects, 1990-2021: an analysis of the Global Burden of Disease Study 2021 and forecast to 2040. EClinicalMedicine.

[16] Lin L, Liang Y, Jiang G, Gan Q, Yang T, Liao P (2025). Global, regional, and national burden of cataract: a comprehensive analysis and projections from 1990 to 2021. PLoS One.

[17] Riebler A, Held L (2017). Projecting the future burden of cancer: Bayesian age-period-cohort analysis with integrated nested Laplace approximations. Biom J.

[18] Yin B, Zhou H (2025). Burden and trends of ovarian and uterine cancer due to high body mass index from 1990 to 2021: an age-period-cohort study based on the GBD 2021, and projections through 2036. Front Oncol.

[19] GBD 2021 Risk Factors Collaborators (2024). Global burden and strength of evidence for 88 risk factors in 204 countries and 811 subnational locations, 1990-2021: a systematic analysis for the Global Burden of Disease Study 2021. Lancet.

[20] Li J, Hu Q, Leng J, Zhou B, Chen C, Hu Y (2025). Global burden of metabolic-associated fatty liver disease among women of childbearing age: trends from 1990 to 2021 and projections to 2040. PLoS One.

[21] Allen L, Williams J, Townsend N, Mikkelsen B, Roberts N, Foster C (2017). Socioeconomic status and non-communicable disease behavioural risk factors in low-income and lower-middle-income countries: a systematic review. Lancet Glob Health.

[22] Stalsberg R, Pedersen AV (2018). Are differences in physical activity across socioeconomic groups associated with choice of physical activity variables to report?. Int J Environ Res Public Health.

[23] Ji P, Gong Y, Jin ML, Hu X, Di GH, Shao ZM (2020). The burden and trends of breast cancer from 1990 to 2017 at the global, regional, and national levels: results from the Global Burden of Disease Study 2017. Front Oncol.

[24] Effiong ME, Afolabi IS, Chinedu SN (2024). Addressing knowledge and behavior gaps in breast cancer risks: implications for health promotion and intervention strategies. Front Oncol.

[25] Singleton AC, Partridge SR, Hyun KK, Mitchell C, Raeside R, Hafiz N (2024). Text message intervention delivered from Australian general practices to improve breast cancer survivors’ physical activity and cardiovascular risk factors: protocol for the EMPOWER-SMS-GP effectiveness implementation randomised controlled trial. BMJ Open.

[26] Rufa’i AA, Wan Muda WA, Yen SH, Abd Shatar AK, Murali BV, Tan SW (2016). Design of a randomised intervention study: the effect of dumbbell exercise therapy on physical activity and quality of life among breast cancer survivors in Malaysia. BMJ Glob Health.

[27] Kim MK, Kim Y, Park S, Kim E, Kim Y, Kim Y (2020). Effects of steady low-intensity exercise on high-fat diet stimulated breast cancer progression via the alteration of macrophage polarization. Integr Cancer Ther.

[28] García-Sancha N, Corchado-Cobos R, Pérez-Losada J (2025). Understanding susceptibility to breast cancer: from risk factors to prevention strategies. Int J Mol Sci.

[29] Cadmus-Bertram L, Hartman SJ, Nelson S, Parker BA, Pierce JP (2015). 12-month web-and phone-based weight loss intervention for women at elevated breast cancer risk: 2337 board# 84 May 29, 9. Med Sci Sports Exerc.

[30] Zhao FY, Liu JE, Fang XM, Chen L, Liang JG, Liu Y (2024). Effects of a 12-week exercise-based intervention on weight management in overweight or obese breast cancer survivors: a randomized controlled trial. Support Care Cancer.

[31] Saxton JM, Wilson C (2023). Tackling the adverse health effects of excess body fat in breast cancer: where does physical activity fit in?. Proc Nutr Soc.

[32] Kehm RD, Genkinger JM, MacInnis RJ, John EM, Phillips KA, Dite GS (2020). Recreational physical activity is associated with reduced breast cancer risk in adult women at high risk for breast cancer: a cohort study of women selected for familial and genetic risk. Cancer Res.

