# Supplementary material for: Global, Regional, and National Burden of Breast Cancer Attributable to Low Physical Activity in Women of Reproductive Age: Historical Trends from 1990 to 2021 and Projections to 2035
Source: Arch Iran Med. 2025 Nov 1;28(11):630–41. doi: 10.34172/aim.34998 (PMC12958434; doi:10.34172/aim.34998)
Supplement: Supplementary file 1 — contains Tables S1-S4 and Figures S1-S2. [file aim-28-630-s001.pdf]

Arch Iran Med. November 2025;28(11):630-S1

10.34172/aim.34998

http://journalaim.com

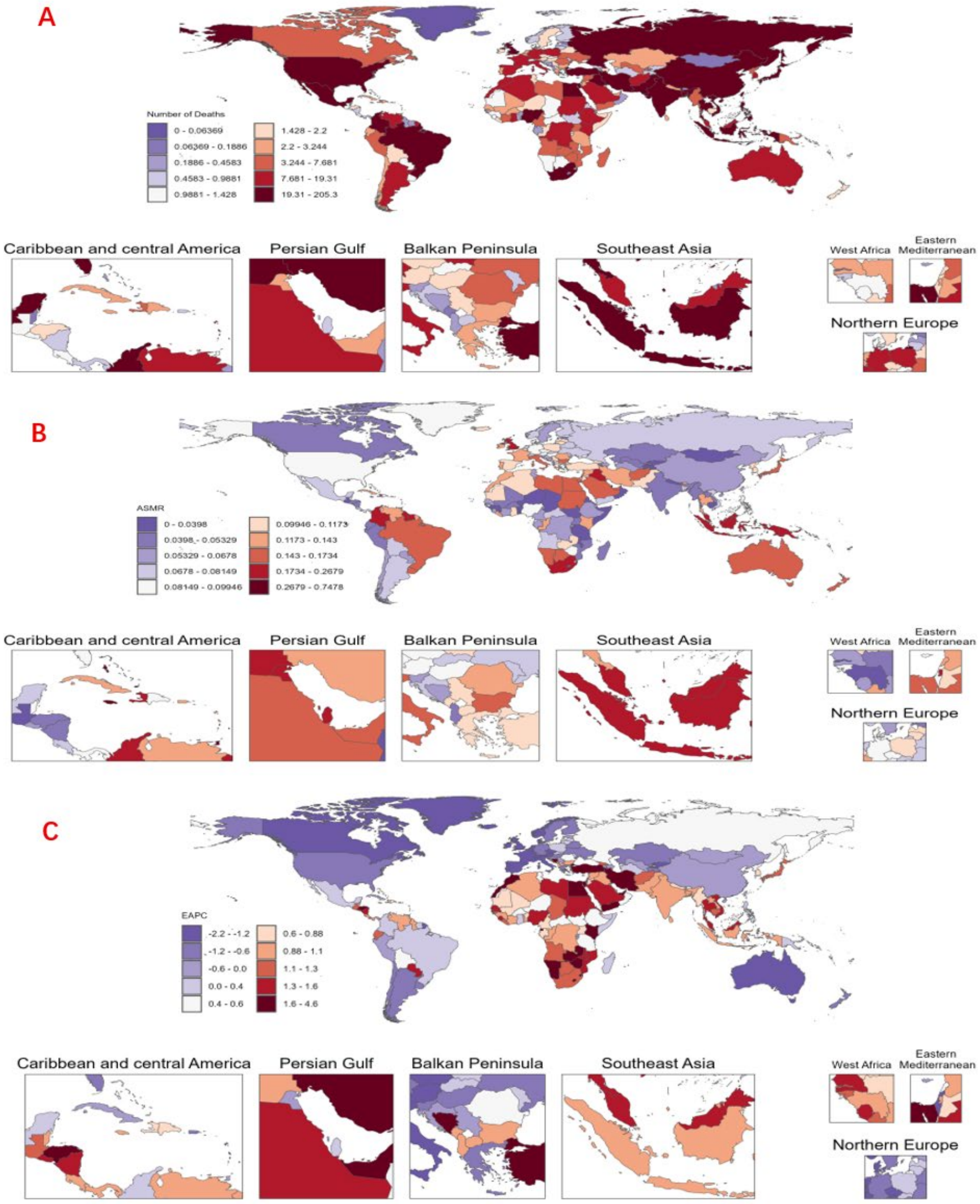

Supplementary Figure 1. Global distribution and trends of breast cancer burden associated with physical inactivity among women of reproductive age in 204 countries. Maps illustrate the deaths (A) and ASMR (B) from breast cancer attributable to low physical activity among women of reproductive age across 204 countries in 2021. EAPCs in ASMR (C) from attributable to low physical activity among women of reproductive age in 204 countries from 1990 to 2021. ASMR, age-standardized mortality rate; EAPC, Estimated Annual Percentage

Change.

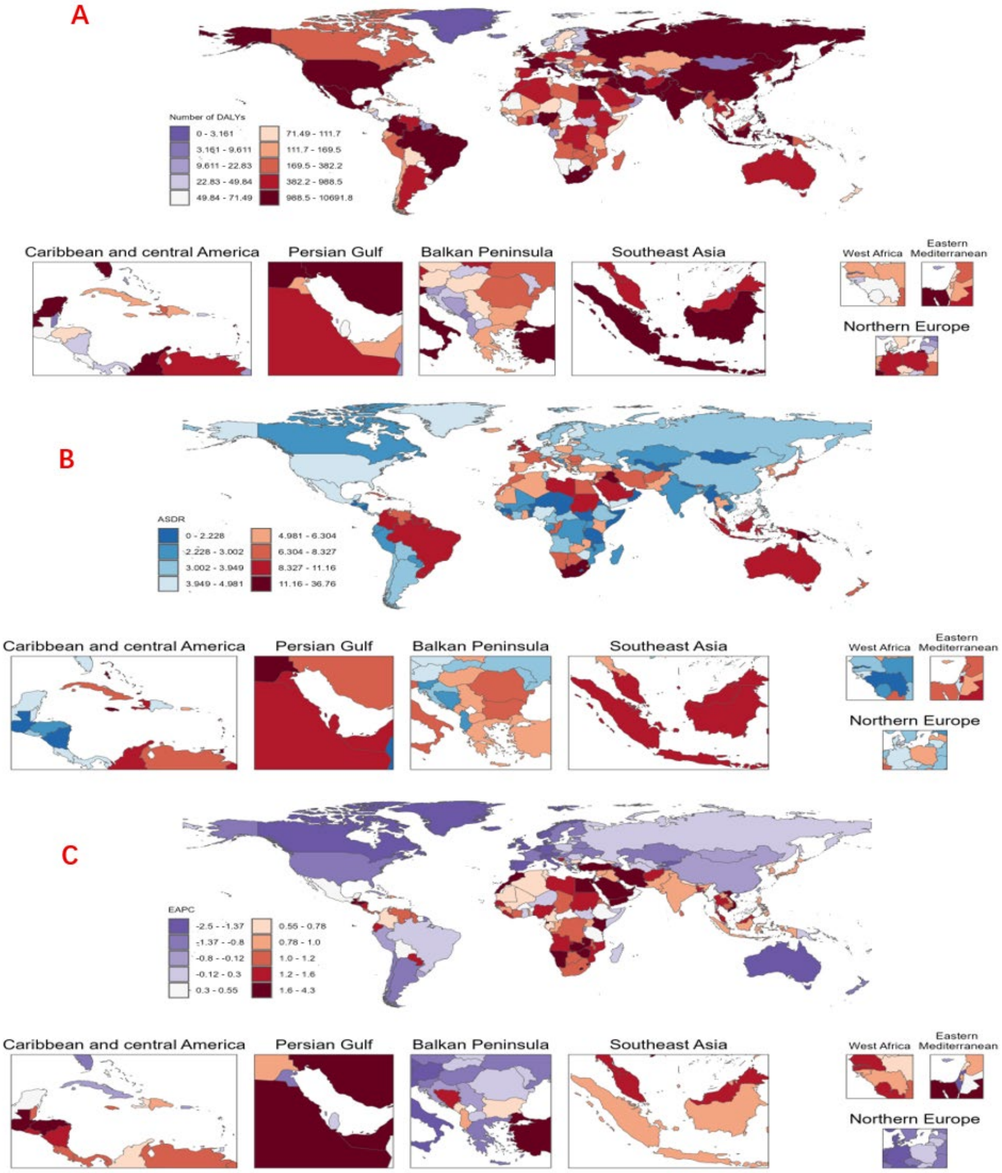

Supplementary Figure 2. Global distribution and trends of breast cancer burden associated with physical inactivity among women of reproductive age in 204 countries. Maps illustrate the DALYs (A) and ASDR (B) from breast cancer attributable to low physical activity among women of reproductive age across 204 countries in 2021. EAPCs in ASDR (C) from attributable to low physical activity among women of reproductive age in 204 countries from 1990 to 2021. DALYs, disability-adjusted life years; ASDR, age-standardized DALY rate; EAPC, Estimated Annual Percentage Change.

Supplementary Table 1

Characteristics	1990		2021		1990-2021
	Number of Deaths (95% UI)	Age-standardized Death rate/100000 (95% UI)	Number of Deaths (95% UI)	Age-standardized Death rate/100000 (95% UI)	EAPC (95% CI)
21 GBD region					
Andean Latin America	4 (1, 8)	0.04 (0.01, 0.09)	9 (2, 20)	0.05 (0.01, 0.11)	0.40 (0.25, 0.55)
Australasia	13 (3, 24)	0.24 (0.05, 0.45)	11 (2, 21)	0.16 (0.03, 0.29)	-1.51 (-1.62, -1.41)
Caribbean	12 (2, 21)	0.13 (0.03, 0.23)	20 (4, 38)	0.17 (0.03, 0.31)	1.00 (0.91, 1.10)
Central Asia	9 (2, 16)	0.05 (0.01, 0.10)	11 (2, 21)	0.05 (0.01, 0.09)	-0.44 (-0.67, -0.22)
Central Europe	38 (7, 67)	0.12 (0.02, 0.22)	28 (6, 51)	0.11 (0.02, 0.20)	-0.87 (-1.10, -0.63)
Central Latin America	25 (5, 45)	0.06 (0.01, 0.11)	67 (13, 122)	0.10 (0.02, 0.18)	1.72 (1.44, 2.00)
Central Sub-Saharan Africa	6 (1, 12)	0.05 (0.01, 0.10)	22 (4, 45)	0.07 (0.01, 0.14)	1.18 (0.97, 1.39)
East Asia	161 (29, 315)	0.05 (0.01, 0.09)	223 (36, 478)	0.07 (0.01, 0.14)	0.81 (0.69, 0.92)
Eastern Europe	44 (8, 83)	0.08 (0.01, 0.15)	35 (6, 69)	0.07 (0.01, 0.14)	-1.19 (-1.52, -0.86)
Eastern Sub-Saharan Africa	17 (3, 32)	0.04 (0.01, 0.08)	60 (11, 112)	0.06 (0.01, 0.10)	1.14 (0.94, 1.33)
High-income Asia Pacific	56 (11, 103)	0.12 (0.02, 0.22)	53 (10, 98)	0.14 (0.03, 0.26)	0.25 (0.11, 0.39)
High-income North America	98 (21, 189)	0.13 (0.03, 0.25)	73 (13, 141)	0.09 (0.02, 0.17)	-1.42 (-1.54, -1.30)
North Africa and Middle East	60 (12, 112)	0.08 (0.02, 0.14)	228 (48, 408)	0.14 (0.03, 0.26)	2.24 (2.11, 2.37)
Oceania	3 (1, 6)	0.20 (0.04, 0.40)	9 (2, 18)	0.26 (0.05, 0.52)	0.83 (0.73, 0.92)
South Asia	107 (22, 198)	0.04 (0.01, 0.08)	299 (60, 575)	0.06 (0.01, 0.12)	0.97 (0.75, 1.18)
Southeast Asia	84 (16, 170)	0.07 (0.01, 0.14)	232 (44, 444)	0.13 (0.02, 0.24)	2.05 (2.01, 2.08)
Southern Latin America	13 (2, 27)	0.11 (0.02, 0.22)	13 (2, 27)	0.08 (0.01, 0.15)	-1.16 (-1.36, -0.95)
Southern	18 (4,	0.14 (0.03, 0.24)	42 (9, 75)	0.19 (0.04, 0.35)	1.75 (1.32,

Sub-Saharan Africa	32)				2.18)
Tropical Latin America	40 (8, 77)	0.10 (0.02, 0.19)	100 (19, 193)	0.16 (0.03, 0.32)	1.48 (1.42, 1.54)
Western Europe	207 (40, 365)	0.22 (0.04, 0.38)	118 (23, 210)	0.13 (0.02, 0.23)	-1.80 (-1.89, -1.71)
Western Sub-Saharan Africa	24 (4, 46)	0.05 (0.01, 0.10)	91 (17, 178)	0.08 (0.01, 0.15)	1.25 (1.08, 1.42)
Country					
Afghanistan	2 (0, 7)	0.11 (0.01, 0.30)	11 (2, 28)	0.15 (0.02, 0.38)	2.06 (1.47, 2.65)
Albania	0 (0, 1)	0.03 (0.01, 0.08)	0 (0, 1)	0.05 (0.01, 0.13)	1.77 (1.30, 2.25)
Algeria	3 (1, 7)	0.06 (0.01, 0.12)	12 (2, 23)	0.10 (0.02, 0.20)	1.84 (1.79, 1.89)
American Samoa	0 (0, 0)	0.32 (0.06, 0.60)	0 (0, 0)	0.75 (0.14, 1.42)	3.16 (2.98, 3.34)
Andorra	0 (0, 0)	0.15 (0.03, 0.32)	0 (0, 0)	0.15 (0.02, 0.35)	0.49 (0.29, 0.70)
Angola	1 (0, 2)	0.05 (0.01, 0.11)	5 (1, 12)	0.07 (0.01, 0.16)	1.42 (1.25, 1.59)
Antigua and Barbuda	0 (0, 0)	0.15 (0.03, 0.31)	0 (0, 0)	0.20 (0.04, 0.40)	1.50 (1.28, 1.73)
Argentine Republic	9 (1, 20)	0.11 (0.02, 0.25)	9 (1, 19)	0.07 (0.01, 0.16)	-1.57 (-1.86, -1.28)
Armenia	1 (0, 2)	0.09 (0.02, 0.20)	0 (0, 1)	0.04 (0.01, 0.10)	-2.57 (-3.35, -1.78)
Australia	11 (2, 22)	0.25 (0.05, 0.49)	9 (2, 17)	0.16 (0.03, 0.29)	-1.75 (-1.87, -1.63)
Austria	4 (1, 7)	0.18 (0.03, 0.35)	2 (0, 4)	0.09 (0.02, 0.18)	-2.14 (-2.33, -1.96)
Azerbaijan	1 (0, 2)	0.05 (0.01, 0.12)	2 (0, 3)	0.05 (0.01, 0.12)	-0.04 (-0.42, 0.34)
Bahamas	0 (0, 0)	0.25 (0.04, 0.49)	0 (0, 1)	0.36 (0.07, 0.78)	1.31 (1.07, 1.54)
Bahrain	0 (0, 0)	0.15 (0.03, 0.30)	1 (0, 1)	0.22 (0.04, 0.44)	1.06 (0.68, 1.45)
Bangladesh	8 (1, 18)	0.03 (0.01, 0.07)	33 (5, 71)	0.07 (0.01, 0.15)	3.01 (2.69, 3.33)
Barbados	0 (0, 0)	0.38 (0.07, 0.69)	0 (0, 1)	0.46 (0.09, 0.88)	0.72 (0.37, 1.07)
Belarus	2 (0, 5)	0.09 (0.01, 0.19)	2 (0, 4)	0.08 (0.01, 0.18)	-1.49 (-1.91, -1.08)

Belgium	6 (1, 12)	0.26 (0.05, 0.51)	3 (1, 6)	0.12 (0.02, 0.24)	-3.04 (-3.34, -2.75)
Belize	0 (0, 0)	0.05 (0.01, 0.09)	0 (0, 0)	0.09 (0.02, 0.18)	2.50 (2.11, 2.89)
Benin	0 (0, 0)	0.01 (0.00, 0.03)	0 (0, 1)	0.01 (0.00, 0.03)	0.23 (-0.15, 0.62)
Bermuda	0 (0, 0)	0.21 (0.04, 0.41)	0 (0, 0)	0.17 (0.03, 0.34)	-1.27 (-1.43, -1.12)
Bhutan	0 (0, 0)	0.09 (0.02, 0.19)	0 (0, 1)	0.14 (0.02, 0.28)	0.74 (0.56, 0.92)
Bolivarian Republic of Venezuela	3 (0, 6)	0.06 (0.01, 0.12)	9 (1, 19)	0.12 (0.02, 0.28)	1.85 (1.58, 2.12)
Bosnia and Herzegovina	1 (0, 1)	0.05 (0.01, 0.12)	0 (0, 1)	0.06 (0.01, 0.13)	0.37 (0.09, 0.65)
Botswana	0 (0, 1)	0.09 (0.02, 0.21)	1 (0, 2)	0.15 (0.02, 0.34)	1.94 (1.51, 2.38)
Brazil	39 (8, 77)	0.10 (0.02, 0.20)	99 (19, 192)	0.17 (0.03, 0.33)	1.50 (1.44, 1.56)
Brunei Darussalam	0 (0, 0)	0.14 (0.02, 0.29)	0 (0, 1)	0.20 (0.03, 0.41)	1.64 (1.31, 1.97)
Bulgaria	3 (1, 6)	0.16 (0.02, 0.31)	2 (0, 5)	0.16 (0.03, 0.34)	-0.05 (-0.31, 0.21)
Burkina Faso	1 (0, 2)	0.05 (0.01, 0.10)	2 (0, 5)	0.04 (0.01, 0.10)	-0.01 (-0.29, 0.27)
Burundi	0 (0, 1)	0.04 (0.01, 0.09)	1 (0, 3)	0.03 (0.00, 0.08)	-1.04 (-1.32, -0.75)
Cabo Verde	0 (0, 0)	0.05 (0.01, 0.11)	0 (0, 0)	0.06 (0.01, 0.12)	0.32 (0.12, 0.53)
Cambodia	1 (0, 1)	0.02 (0.00, 0.05)	2 (0, 4)	0.03 (0.01, 0.09)	1.29 (1.09, 1.48)
Cameroon	1 (0, 3)	0.05 (0.01, 0.11)	5 (1, 12)	0.07 (0.01, 0.16)	0.76 (0.61, 0.92)
Canada	6 (1, 13)	0.08 (0.01, 0.17)	4 (1, 10)	0.05 (0.01, 0.12)	-1.61 (-1.73, -1.50)
Central African Republic	0 (0, 1)	0.06 (0.01, 0.15)	1 (0, 2)	0.08 (0.01, 0.18)	0.54 (0.40, 0.68)
Chad	0 (0, 1)	0.03 (0.00, 0.06)	1 (0, 3)	0.03 (0.00, 0.08)	0.95 (0.80, 1.10)
Chile	3 (1, 6)	0.08 (0.02, 0.16)	3 (1, 7)	0.07 (0.01, 0.14)	-0.27 (-0.43, -0.11)
China	151 (27, 300)	0.05 (0.01, 0.09)	205 (32, 445)	0.06 (0.01, 0.14)	0.75 (0.63, 0.87)
Colombia	10 (2,	0.11 (0.02, 0.22)	24 (4, 46)	0.18 (0.03, 0.35)	2.10 (1.66,

	19)				2.55)
Comoros	0 (0, 0)	0.04 (0.00, 0.09)	0 (0, 0)	0.08 (0.01, 0.20)	2.99 (2.50, 3.49)
Cook Islands	0 (0, 0)	0.36 (0.07, 0.73)	0 (0, 0)	0.41 (0.08, 0.85)	1.05 (0.83, 1.27)
Costa Rica	0 (0, 1)	0.05 (0.01, 0.11)	1 (0, 2)	0.08 (0.01, 0.17)	1.60 (1.41, 1.79)
Croatia	1 (0, 2)	0.09 (0.02, 0.19)	1 (0, 2)	0.08 (0.01, 0.17)	-0.63 (-0.88, -0.38)
Cuba	4 (1, 8)	0.12 (0.02, 0.25)	3 (0, 7)	0.13 (0.02, 0.26)	0.37 (0.13, 0.60)
Cyprus	0 (0, 1)	0.13 (0.02, 0.27)	0 (0, 1)	0.10 (0.01, 0.21)	-1.24 (-1.46, -1.03)
Czech Republic	3 (0, 5)	0.10 (0.02, 0.20)	2 (0, 4)	0.09 (0.02, 0.18)	-0.85 (-1.23, -0.47)
Democratic People's Republic of Korea	4 (1, 9)	0.07 (0.01, 0.16)	7 (1, 16)	0.11 (0.02, 0.24)	1.72 (1.56, 1.89)
Democratic Republic of the Congo	4 (1, 8)	0.04 (0.01, 0.10)	13 (2, 30)	0.06 (0.01, 0.14)	1.03 (0.71, 1.35)
Denmark	3 (1, 7)	0.25 (0.04, 0.50)	1 (0, 2)	0.08 (0.01, 0.18)	-3.67 (-3.78, -3.55)
Djibouti	0 (0, 0)	0.04 (0.01, 0.11)	0 (0, 1)	0.07 (0.01, 0.17)	1.79 (1.63, 1.96)
Dominica	0 (0, 0)	0.11 (0.02, 0.25)	0 (0, 0)	0.17 (0.03, 0.34)	1.68 (1.54, 1.82)
Dominican Republic	1 (0, 2)	0.06 (0.01, 0.13)	3 (0, 6)	0.10 (0.02, 0.20)	1.66 (1.43, 1.89)
Ecuador	1 (0, 2)	0.03 (0.01, 0.06)	2 (0, 5)	0.05 (0.01, 0.11)	1.59 (1.37, 1.81)
Egypt	16 (3, 31)	0.12 (0.02, 0.24)	41 (9, 82)	0.16 (0.03, 0.32)	0.56 (0.40, 0.72)
El Salvador	0 (0, 1)	0.04 (0.01, 0.07)	1 (0, 3)	0.07 (0.01, 0.14)	2.09 (1.97, 2.22)
Equatorial Guinea	0 (0, 0)	0.05 (0.01, 0.11)	0 (0, 1)	0.08 (0.01, 0.22)	1.71 (1.57, 1.85)
Eritrea	0 (0, 1)	0.02 (0.00, 0.06)	1 (0, 2)	0.04 (0.01, 0.10)	1.66 (1.49, 1.83)
Estonia	0 (0, 1)	0.08 (0.01, 0.16)	0 (0, 0)	0.05 (0.01, 0.12)	-1.92 (-2.26, -1.58)
Eswatini	0 (0, 0)	0.06 (0.01, 0.13)	0 (0, 1)	0.13 (0.02, 0.33)	2.83 (2.40, 3.27)
Ethiopia	6 (1,	0.05 (0.01, 0.13)	17 (3, 33)	0.06 (0.01, 0.12)	0.46 (0.17,

	15)				0.76)
Federated States of Micronesia	0 (0, 0)	0.38 (0.08, 0.81)	0 (0, 0)	0.58 (0.11, 1.19)	1.37 (1.23, 1.50)
Fiji	1 (0, 1)	0.33 (0.06, 0.68)	1 (0, 2)	0.42 (0.09, 0.86)	1.01 (0.82, 1.20)
Finland	2 (0, 5)	0.17 (0.03, 0.36)	1 (0, 2)	0.07 (0.01, 0.16)	-3.32 (-3.56, -3.07)
French Republic	28 (5, 55)	0.20 (0.03, 0.38)	19 (3, 37)	0.13 (0.02, 0.26)	-1.47 (-1.75, -1.19)
Gabonese Republic	0 (0, 0)	0.04 (0.01, 0.09)	0 (0, 1)	0.06 (0.01, 0.14)	1.13 (0.94, 1.31)
Gambia	0 (0, 0)	0.02 (0.00, 0.03)	0 (0, 0)	0.03 (0.01, 0.07)	1.72 (1.40, 2.04)
Georgia	2 (0, 3)	0.12 (0.02, 0.24)	1 (0, 2)	0.10 (0.01, 0.22)	-0.42 (-0.71, -0.13)
Germany	30 (5, 66)	0.15 (0.03, 0.34)	15 (2, 31)	0.09 (0.01, 0.18)	-1.89 (-2.03, -1.74)
Ghana	3 (0, 6)	0.09 (0.01, 0.18)	9 (2, 21)	0.10 (0.02, 0.23)	0.39 (-0.03, 0.82)
Greenland	0 (0, 0)	0.13 (0.02, 0.29)	0 (0, 0)	0.08 (0.01, 0.21)	-1.23 (-1.70, -0.75)
Grenada	0 (0, 0)	0.17 (0.03, 0.35)	0 (0, 0)	0.22 (0.04, 0.44)	1.20 (0.85, 1.55)
Guam	0 (0, 0)	0.06 (0.01, 0.13)	0 (0, 0)	0.12 (0.02, 0.26)	2.50 (2.08, 2.93)
Guatemala	0 (0, 0)	0.01 (0.00, 0.03)	1 (0, 2)	0.02 (0.00, 0.05)	2.35 (1.81, 2.89)
Guinea-Bissau	0 (0, 0)	0.06 (0.01, 0.14)	0 (0, 1)	0.09 (0.01, 0.20)	0.89 (0.76, 1.03)
Guyana	0 (0, 0)	0.12 (0.02, 0.23)	0 (0, 1)	0.21 (0.04, 0.44)	2.62 (2.07, 3.17)
Haiti	2 (0, 5)	0.14 (0.02, 0.34)	6 (1, 14)	0.18 (0.03, 0.40)	0.86 (0.77, 0.94)
Hellenic Republic	3 (0, 6)	0.12 (0.02, 0.25)	2 (0, 5)	0.10 (0.02, 0.23)	-0.66 (-0.97, -0.35)
Honduras	0 (0, 1)	0.03 (0.01, 0.08)	1 (0, 4)	0.05 (0.01, 0.13)	1.08 (0.95, 1.21)
Hungary	3 (1, 7)	0.13 (0.02, 0.28)	2 (0, 4)	0.10 (0.01, 0.20)	-1.39 (-1.60, -1.17)
Iceland	0 (0, 0)	0.15 (0.02, 0.30)	0 (0, 0)	0.11 (0.02, 0.22)	-1.73 (-1.99, -1.47)
India	80 (16, 156)	0.04 (0.01, 0.08)	193 (38, 392)	0.05 (0.01, 0.10)	0.58 (0.33, 0.83)

Indonesia	50 (10, 108)	0.10 (0.02, 0.23)	133 (23, 278)	0.18 (0.03, 0.37)	1.85 (1.80, 1.90)
Iran (Islamic Republic of)	9 (2, 16)	0.07 (0.01, 0.13)	33 (6, 61)	0.14 (0.02, 0.26)	2.72 (2.25, 3.19)
Iraq	6 (1, 10)	0.14 (0.03, 0.26)	24 (5, 49)	0.23 (0.05, 0.47)	1.51 (1.38, 1.65)
Ireland	2 (0, 4)	0.23 (0.04, 0.46)	2 (0, 3)	0.14 (0.02, 0.28)	-1.71 (-1.88, -1.55)
Israel	2 (0, 4)	0.18 (0.03, 0.36)	2 (0, 4)	0.09 (0.02, 0.19)	-2.37 (-2.58, -2.16)
Italy	33 (6, 63)	0.23 (0.04, 0.44)	19 (3, 37)	0.15 (0.03, 0.31)	-1.21 (-1.34, -1.09)
Jamaica	1 (0, 2)	0.17 (0.03, 0.30)	3 (1, 6)	0.40 (0.08, 0.81)	2.91 (2.44, 3.39)
Japan	47 (9, 87)	0.15 (0.03, 0.27)	39 (7, 76)	0.16 (0.03, 0.31)	0.03 (-0.14, 0.20)
Jordan	1 (0, 1)	0.08 (0.02, 0.18)	3 (1, 7)	0.10 (0.02, 0.21)	0.71 (0.43, 0.99)
Kazakhstan	3 (1, 6)	0.07 (0.01, 0.14)	2 (0, 6)	0.05 (0.01, 0.12)	-1.07 (-1.51, -0.63)
Kenya	3 (0, 6)	0.05 (0.01, 0.12)	16 (3, 32)	0.12 (0.02, 0.24)	2.65 (2.58, 2.72)
Kiribati	0 (0, 0)	0.31 (0.06, 0.61)	0 (0, 0)	0.47 (0.09, 0.95)	1.38 (1.22, 1.54)
Kuwait	1 (0, 1)	0.13 (0.03, 0.23)	3 (1, 5)	0.18 (0.04, 0.35)	-0.51 (-1.34, 0.32)
Kyrgyz Republic	1 (0, 1)	0.05 (0.01, 0.11)	1 (0, 1)	0.04 (0.01, 0.09)	-1.33 (-1.58, -1.07)
Lao People's Democratic Republic	0 (0, 1)	0.03 (0.00, 0.09)	1 (0, 3)	0.05 (0.01, 0.14)	1.40 (1.21, 1.59)
Latvia	1 (0, 2)	0.14 (0.03, 0.30)	0 (0, 1)	0.10 (0.02, 0.21)	-1.48 (-1.72, -1.24)
Lebanese Republic	1 (0, 2)	0.11 (0.02, 0.25)	2 (0, 4)	0.14 (0.03, 0.28)	0.72 (0.54, 0.90)
Lesotho	0 (0, 0)	0.02 (0.00, 0.05)	0 (0, 0)	0.03 (0.00, 0.09)	2.46 (2.01, 2.91)
Liberia	0 (0, 1)	0.07 (0.01, 0.14)	2 (0, 4)	0.14 (0.02, 0.29)	1.71 (1.36, 2.05)
Libya	1 (0, 1)	0.07 (0.01, 0.14)	3 (1, 7)	0.17 (0.03, 0.35)	3.56 (3.41, 3.72)
Lithuania	1 (0, 2)	0.11 (0.02, 0.22)	1 (0, 1)	0.10 (0.02, 0.21)	-0.15 (-0.41, 0.11)
Luxembourg	0 (0,	0.19 (0.04, 0.39)	0 (0, 0)	0.08 (0.02, 0.18)	-2.67 (-2.92,

	0)				-2.43)
Madagascar	1 (0, 3)	0.04 (0.01, 0.09)	4 (1, 9)	0.05 (0.01, 0.12)	0.65 (0.48, 0.82)
Malawi	0 (0, 1)	0.02 (0.00, 0.04)	1 (0, 3)	0.02 (0.00, 0.06)	0.84 (0.35, 1.33)
Malaysia	6 (1, 11)	0.12 (0.02, 0.24)	18 (3, 38)	0.22 (0.04, 0.46)	1.85 (1.64, 2.06)
Maldives	0 (0, 0)	0.11 (0.01, 0.28)	0 (0, 0)	0.13 (0.03, 0.25)	0.47 (0.34, 0.59)
Malta	0 (0, 1)	0.27 (0.05, 0.55)	0 (0, 0)	0.21 (0.04, 0.39)	-1.62 (-1.89, -1.35)
Marshall Islands	0 (0, 0)	0.31 (0.06, 0.65)	0 (0, 0)	0.64 (0.12, 1.41)	2.22 (2.05, 2.40)
Mauritania	0 (0, 1)	0.07 (0.01, 0.15)	1 (0, 2)	0.11 (0.02, 0.23)	1.29 (1.16, 1.42)
Mauritius	0 (0, 0)	0.04 (0.01, 0.09)	0 (0, 1)	0.14 (0.02, 0.30)	2.78 (2.33, 3.24)
Monaco	0 (0, 0)	0.26 (0.05, 0.55)	0 (0, 0)	0.28 (0.05, 0.61)	0.33 (0.10, 0.56)
Mongolia	0 (0, 0)	0.01 (0.00, 0.03)	0 (0, 0)	0.02 (0.00, 0.04)	1.13 (0.95, 1.32)
Montenegro	0 (0, 0)	0.11 (0.02, 0.23)	0 (0, 0)	0.11 (0.02, 0.25)	0.54 (0.10, 0.98)
Morocco	4 (1, 8)	0.06 (0.01, 0.12)	14 (2, 29)	0.14 (0.02, 0.30)	2.96 (2.84, 3.07)
Mozambique	1 (0, 3)	0.03 (0.00, 0.08)	3 (1, 9)	0.04 (0.01, 0.11)	1.26 (1.03, 1.50)
Myanmar	3 (0, 8)	0.03 (0.00, 0.08)	6 (1, 16)	0.04 (0.01, 0.11)	0.92 (0.67, 1.18)
Namibia	0 (0, 1)	0.07 (0.02, 0.15)	1 (0, 2)	0.16 (0.03, 0.36)	2.68 (2.60, 2.77)
Nauru	0 (0, 0)	0.34 (0.05, 0.77)	0 (0, 0)	0.41 (0.08, 0.98)	0.58 (0.42, 0.74)
Nepal	1 (0, 2)	0.02 (0.00, 0.05)	3 (0, 7)	0.03 (0.01, 0.08)	1.01 (0.58, 1.44)
Netherlands	6 (1, 13)	0.15 (0.03, 0.33)	3 (0, 6)	0.07 (0.01, 0.16)	-2.63 (-2.85, -2.42)
New Zealand	2 (0, 3)	0.18 (0.03, 0.35)	2 (0, 3)	0.15 (0.03, 0.28)	0.00 (-0.20, 0.21)
Nicaragua	0 (0, 0)	0.03 (0.00, 0.05)	1 (0, 2)	0.04 (0.01, 0.10)	1.75 (1.57, 1.92)
Niger	1 (0, 1)	0.03 (0.01, 0.07)	2 (0, 4)	0.03 (0.00, 0.07)	-0.18 (-0.25, -0.10)

Nigeria	13 (2, 27)	0.06 (0.01, 0.13)	54 (9, 123)	0.09 (0.02, 0.21)	1.55 (1.39, 1.72)
Niue	0 (0, 0)	0.31 (0.05, 0.61)	0 (0, 0)	0.41 (0.08, 0.82)	0.59 (0.47, 0.71)
North Macedonia	1 (0, 1)	0.13 (0.02, 0.27)	1 (0, 1)	0.10 (0.02, 0.24)	-1.00 (-1.28, -0.71)
Northern Mariana Islands	0 (0, 0)	0.23 (0.04, 0.48)	0 (0, 0)	0.36 (0.08, 0.70)	1.81 (1.29, 2.34)
Norway	2 (0, 3)	0.15 (0.03, 0.30)	1 (0, 2)	0.07 (0.01, 0.15)	-2.86 (-3.11, -2.61)
Oman	0 (0, 0)	0.03 (0.00, 0.06)	0 (0, 1)	0.03 (0.01, 0.07)	0.50 (0.35, 0.65)
Pakistan	18 (3, 37)	0.07 (0.01, 0.15)	70 (12, 148)	0.12 (0.02, 0.24)	0.94 (0.77, 1.11)
Palau	0 (0, 0)	0.35 (0.07, 0.71)	0 (0, 0)	0.50 (0.09, 1.02)	0.96 (0.86, 1.06)
Palestine	1 (0, 2)	0.18 (0.03, 0.36)	3 (0, 5)	0.21 (0.04, 0.38)	0.61 (0.52, 0.71)
Panama	0 (0, 1)	0.05 (0.01, 0.10)	1 (0, 2)	0.08 (0.01, 0.17)	2.22 (2.02, 2.42)
Papua New Guinea	2 (0, 4)	0.18 (0.03, 0.40)	6 (1, 14)	0.24 (0.04, 0.52)	0.86 (0.71, 1.01)
Paraguay	0 (0, 1)	0.04 (0.01, 0.09)	1 (0, 3)	0.05 (0.01, 0.14)	1.06 (0.96, 1.16)
Peru	3 (0, 6)	0.05 (0.01, 0.10)	5 (1, 12)	0.05 (0.01, 0.13)	-0.11 (-0.33, 0.10)
Philippines	9 (1, 19)	0.06 (0.01, 0.12)	29 (5, 63)	0.10 (0.02, 0.21)	2.03 (1.86, 2.21)
Plurinational State of Bolivia	1 (0, 2)	0.05 (0.01, 0.13)	2 (0, 5)	0.07 (0.01, 0.16)	0.49 (0.37, 0.61)
Poland	12 (3, 24)	0.13 (0.03, 0.26)	10 (2, 20)	0.11 (0.02, 0.22)	-1.33 (-1.65, -1.01)
Portuguese Republic	5 (1, 9)	0.18 (0.03, 0.36)	3 (0, 6)	0.14 (0.02, 0.28)	-0.80 (-0.97, -0.63)
Puerto Rico	1 (0, 3)	0.15 (0.03, 0.30)	1 (0, 2)	0.12 (0.03, 0.25)	-0.92 (-1.13, -0.70)
Qatar	0 (0, 0)	0.23 (0.04, 0.46)	1 (0, 2)	0.18 (0.03, 0.35)	-1.11 (-1.49, -0.72)
Republic of Côte d'Ivoire	1 (0, 2)	0.04 (0.01, 0.08)	4 (1, 9)	0.06 (0.01, 0.14)	2.04 (1.82, 2.26)
Republic of Guinea	0 (0, 1)	0.03 (0.00, 0.07)	1 (0, 3)	0.04 (0.01, 0.09)	0.54 (0.37, 0.71)
Republic of Korea	8 (2, 15)	0.06 (0.01, 0.12)	12 (2, 25)	0.11 (0.02, 0.22)	1.86 (1.61, 2.11)

Republic of Mali	1 (0, 2)	0.05 (0.01, 0.10)	3 (0, 7)	0.05 (0.01, 0.13)	-0.02 (-0.18, 0.14)
Republic of Moldova	1 (0, 2)	0.09 (0.02, 0.20)	1 (0, 1)	0.07 (0.01, 0.16)	-1.44 (-1.76, -1.12)
Republic of the Congo	0 (0, 1)	0.07 (0.01, 0.19)	2 (0, 4)	0.13 (0.02, 0.30)	1.92 (1.62, 2.22)
Romania	8 (2, 16)	0.14 (0.03, 0.28)	6 (1, 12)	0.14 (0.03, 0.29)	-0.18 (-0.47, 0.11)
Russian Federation	26 (4, 51)	0.07 (0.01, 0.14)	24 (4, 50)	0.07 (0.01, 0.15)	-0.80 (-1.16, -0.44)
Rwanda	1 (0, 2)	0.05 (0.01, 0.13)	2 (0, 5)	0.05 (0.01, 0.14)	-0.79 (-1.18, -0.39)
Saint Kitts and Nevis	0 (0, 0)	0.23 (0.04, 0.46)	0 (0, 0)	0.17 (0.03, 0.36)	-1.10 (-1.30, -0.90)
Saint Lucia	0 (0, 0)	0.16 (0.03, 0.33)	0 (0, 0)	0.23 (0.04, 0.46)	1.63 (1.29, 1.97)
Saint Vincent and the Grenadines	0 (0, 0)	0.17 (0.03, 0.35)	0 (0, 0)	0.28 (0.05, 0.62)	1.77 (1.57, 1.97)
Samoa	0 (0, 0)	0.21 (0.05, 0.40)	0 (0, 0)	0.33 (0.06, 0.65)	1.71 (1.56, 1.86)
San Marino	0 (0, 0)	0.10 (0.02, 0.21)	0 (0, 0)	0.09 (0.02, 0.22)	0.82 (0.40, 1.25)
Sao Tome and Principe	0 (0, 0)	0.03 (0.00, 0.07)	0 (0, 0)	0.06 (0.01, 0.14)	2.17 (2.00, 2.34)
Saudi Arabia	2 (0, 5)	0.06 (0.01, 0.14)	17 (3, 34)	0.16 (0.03, 0.33)	3.17 (3.11, 3.22)
Senegal	1 (0, 1)	0.04 (0.01, 0.08)	2 (0, 5)	0.06 (0.01, 0.14)	1.57 (1.37, 1.77)
Serbia	3 (0, 6)	0.13 (0.02, 0.27)	2 (0, 5)	0.11 (0.02, 0.23)	-1.28 (-1.65, -0.91)
Seychelles	0 (0, 0)	0.06 (0.01, 0.13)	0 (0, 0)	0.13 (0.02, 0.27)	3.14 (2.71, 3.56)
Sierra Leone	0 (0, 1)	0.03 (0.00, 0.08)	1 (0, 3)	0.05 (0.01, 0.12)	1.74 (1.66, 1.81)
Singapore	1 (0, 2)	0.13 (0.02, 0.26)	1 (0, 3)	0.09 (0.02, 0.18)	-1.54 (-1.80, -1.28)
Slovak Republic	1 (0, 3)	0.10 (0.02, 0.20)	1 (0, 2)	0.08 (0.01, 0.18)	-1.13 (-1.35, -0.91)
Slovenia	0 (0, 1)	0.10 (0.02, 0.20)	0 (0, 0)	0.05 (0.01, 0.12)	-1.91 (-2.21, -1.61)
Solomon Islands	0 (0, 0)	0.13 (0.02, 0.27)	1 (0, 1)	0.29 (0.06, 0.61)	3.09 (2.94, 3.24)
Somalia	1 (0, 2)	0.03 (0.01, 0.09)	2 (0, 4)	0.03 (0.00, 0.08)	-0.79 (-1.05, -0.53)

South Africa	17 (3, 30)	0.17 (0.04, 0.32)	35 (8, 63)	0.23 (0.05, 0.41)	1.53 (1.09, 1.97)
South Sudan	0 (0, 1)	0.03 (0.00, 0.07)	1 (0, 2)	0.04 (0.01, 0.09)	1.19 (0.71, 1.68)
Spain	16 (3, 33)	0.17 (0.03, 0.34)	11 (2, 25)	0.12 (0.02, 0.25)	-0.99 (-1.17, -0.82)
Sri Lanka	1 (0, 3)	0.03 (0.01, 0.07)	3 (0, 6)	0.05 (0.01, 0.11)	1.97 (1.74, 2.21)
Sudan	5 (1, 12)	0.11 (0.02, 0.25)	19 (3, 41)	0.17 (0.03, 0.37)	1.64 (1.43, 1.86)
Suriname	0 (0, 0)	0.13 (0.02, 0.25)	0 (0, 1)	0.19 (0.04, 0.38)	1.41 (1.24, 1.58)
Sweden	3 (1, 7)	0.15 (0.03, 0.33)	1 (0, 3)	0.07 (0.01, 0.13)	-2.11 (-2.35, -1.87)
Swiss Confederation	3 (1, 6)	0.15 (0.03, 0.33)	1 (0, 3)	0.07 (0.01, 0.14)	-2.44 (-2.76, -2.12)
Syrian Arab Republic	2 (0, 5)	0.08 (0.01, 0.17)	5 (1, 12)	0.14 (0.03, 0.31)	1.90 (1.47, 2.33)
Taiwan (Province of China)	6 (1, 13)	0.12 (0.02, 0.23)	11 (2, 22)	0.20 (0.04, 0.40)	1.44 (1.11, 1.77)
Tajikistan	0 (0, 1)	0.04 (0.01, 0.08)	1 (0, 2)	0.04 (0.01, 0.10)	-0.19 (-0.34, -0.04)
Thailand	9 (1, 19)	0.05 (0.01, 0.12)	19 (3, 45)	0.12 (0.02, 0.27)	2.57 (2.21, 2.94)
Timor-Leste	0 (0, 0)	0.03 (0.00, 0.06)	0 (0, 0)	0.04 (0.01, 0.09)	1.03 (0.67, 1.40)
Togolese Republic	0 (0, 0)	0.02 (0.00, 0.05)	1 (0, 2)	0.03 (0.00, 0.08)	1.19 (0.86, 1.51)
Tokelau	0 (0, 0)	0.30 (0.05, 0.63)	0 (0, 0)	0.42 (0.08, 0.83)	0.80 (0.66, 0.94)
Tonga	0 (0, 0)	0.34 (0.07, 0.65)	0 (0, 0)	0.42 (0.09, 0.87)	0.72 (0.67, 0.78)
Trinidad and Tobago	1 (0, 1)	0.25 (0.05, 0.48)	1 (0, 3)	0.39 (0.07, 0.80)	1.10 (0.91, 1.29)
Tunisia	1 (0, 1)	0.03 (0.00, 0.06)	2 (0, 4)	0.06 (0.01, 0.13)	2.45 (2.38, 2.52)
Turkey	4 (1, 9)	0.03 (0.01, 0.07)	25 (5, 49)	0.11 (0.02, 0.23)	6.09 (5.13, 7.06)
Turkmenistan	0 (0, 1)	0.04 (0.01, 0.08)	1 (0, 2)	0.06 (0.01, 0.15)	1.95 (1.56, 2.35)
Tuvalu	0 (0, 0)	0.28 (0.05, 0.62)	0 (0, 0)	0.32 (0.06, 0.67)	0.11 (-0.12, 0.34)
Uganda	1 (0, 3)	0.03 (0.00, 0.07)	5 (1, 13)	0.05 (0.01, 0.13)	1.02 (0.69, 1.34)

Ukraine	13 (2, 30)	0.10 (0.02, 0.23)	7 (1, 16)	0.07 (0.01, 0.16)	-2.07 (-2.42, -1.71)
United Arab Emirates	0 (0, 1)	0.10 (0.02, 0.20)	3 (1, 6)	0.17 (0.03, 0.34)	1.21 (0.80, 1.63)
United Kingdom	58 (11, 105)	0.41 (0.08, 0.74)	31 (6, 56)	0.20 (0.04, 0.37)	-2.42 (-2.53, -2.31)
United Mexican States	10 (2, 19)	0.05 (0.01, 0.09)	28 (5, 57)	0.08 (0.02, 0.16)	1.65 (1.37, 1.94)
United Republic of Tanzania	1 (0, 2)	0.02 (0.00, 0.04)	3 (0, 7)	0.02 (0.00, 0.05)	0.39 (0.17, 0.60)
United States of America	92 (20, 182)	0.14 (0.03, 0.27)	68 (12, 135)	0.09 (0.02, 0.18)	-1.41 (-1.54, -1.28)
United States Virgin Islands	0 (0, 0)	0.18 (0.03, 0.39)	0 (0, 0)	0.17 (0.02, 0.39)	0.74 (0.37, 1.11)
Uruguay	1 (0, 3)	0.18 (0.03, 0.39)	1 (0, 3)	0.17 (0.03, 0.35)	-0.24 (-0.39, -0.08)
Uzbekistan	1 (0, 3)	0.03 (0.00, 0.06)	4 (1, 8)	0.04 (0.01, 0.09)	1.82 (1.65, 1.98)
Vanuatu	0 (0, 0)	0.03 (0.00, 0.07)	0 (0, 0)	0.04 (0.01, 0.10)	0.69 (0.43, 0.95)
Viet Nam	5 (1, 13)	0.03 (0.00, 0.08)	20 (3, 43)	0.08 (0.01, 0.17)	3.27 (3.17, 3.38)
Yemen	1 (0, 2)	0.04 (0.01, 0.08)	7 (1, 15)	0.08 (0.02, 0.18)	2.21 (2.02, 2.40)
Zambia	1 (0, 2)	0.05 (0.01, 0.12)	5 (1, 13)	0.10 (0.01, 0.27)	2.64 (2.05, 3.24)
Zimbabwe	1 (0, 2)	0.04 (0.01, 0.08)	4 (1, 9)	0.10 (0.01, 0.22)	4.28 (3.54, 5.02)

Supplementary Table 2

Characteristics	1990		2021		1990-2021
	Number of DALYs (95% UI)	Age-standardized DALYs rate/100000 (95% UI)	Number of DALYs (95% UI)	Age-standardized DALYs rate/100000 (95% UI)	EAPC (95% CI)
21 GBD region					
Andean Latin America	203 (35, 413)	2.14 (0.37, 4.35)	461 (92, 971)	2.64 (0.53, 5.56)	0.44 (0.30, 0.59)
Australasia	651 (133, 1220)	12.12 (2.49, 22.72)	601 (130, 1103)	8.32 (1.80, 15.28)	-1.32 (-1.42, -1.21)
Caribbean	587 (119, 1079)	6.30 (1.28, 11.57)	1011 (200, 1877)	8.41 (1.66, 15.61)	0.97 (0.87, 1.07)
Central Asia	444 (79, 808)	2.65 (0.47, 4.82)	571 (121, 1064)	2.35 (0.50, 4.38)	-0.50 (-0.70, -0.30)
Central Europe	1852 (364, 3349)	6.03 (1.19, 10.90)	1395 (281, 2545)	5.42 (1.09, 9.88)	-0.75 (-0.98, -0.52)
Central Latin America	1255 (255, 2269)	2.99 (0.61, 5.41)	3427 (664, 6228)	5.03 (0.97, 9.13)	1.76 (1.47, 2.05)
Central Sub-Saharan Africa	283 (52, 597)	2.29 (0.42, 4.83)	1074 (212, 2187)	3.29 (0.65, 6.70)	1.18 (0.96, 1.40)
East Asia	8216 (1499, 16030)	2.46 (0.45, 4.81)	11607 (1932, 24729)	3.51 (0.58, 7.47)	0.87 (0.75, 0.98)
Eastern Europe	2162 (388, 4091)	3.91 (0.70, 7.40)	1713 (294, 3382)	3.55 (0.61, 7.01)	-1.15 (-1.45, -0.84)
Eastern Sub-Saharan Africa	828 (148, 1613)	1.92 (0.34, 3.74)	3035 (551, 5665)	2.83 (0.51, 5.29)	1.18 (0.98, 1.38)
High-income Asia Pacific	2906 (561, 5295)	6.35 (1.23, 11.58)	2819 (559, 5148)	7.41 (1.47, 13.53)	0.39 (0.25, 0.53)
High-income North America	5078 (1100, 9754)	6.83 (1.48, 13.12)	3877 (737, 7629)	4.61 (0.88, 9.08)	-1.33 (-1.46, -1.20)
North Africa and Middle East	3035 (611, 5630)	3.89 (0.78, 7.21)	11804 (2505, 21195)	7.41 (1.57, 13.30)	2.32 (2.19, 2.45)
Oceania	159 (31,	10.24 (1.97, 20.19)	454 (84,	13.07 (2.43,	0.79 (0.69,

	314)		899)	25.90)	0.88)
South Asia	5222 (1105, 9730)	2.05 (0.43, 3.82)	14713 (2980, 27991)	2.98 (0.60, 5.66)	1.01 (0.81, 1.21)
Southeast Asia	4039 (778, 8120)	3.36 (0.65, 6.76)	11177 (2138, 21323)	6.10 (1.17, 11.64)	2.04 (2.01, 2.07)
Southern Latin America	638 (117, 1285)	5.15 (0.95, 10.37)	674 (123, 1314)	3.87 (0.71, 7.54)	-1.08 (-1.29, -0.86)
Southern Sub-Saharan Africa	930 (193, 1648)	7.00 (1.45, 12.40)	2071 (463, 3696)	9.54 (2.13, 17.02)	1.64 (1.19, 2.08)
Tropical Latin America	1954 (415, 3782)	4.90 (1.04, 9.48)	4977 (963, 9772)	8.21 (1.59, 16.12)	1.52 (1.46, 1.58)
Western Europe	10424 (2009, 18264)	10.91 (2.10, 19.12)	6229 (1198, 10933)	6.69 (1.29, 11.73)	-1.63 (-1.72, -1.53)
Western Sub-Saharan Africa	1174 (197, 2237)	2.69 (0.45, 5.13)	4508 (857, 8885)	3.76 (0.72, 7.41)	1.25 (1.08, 1.42)
Country					
Afghanistan	114 (15, 315)	5.19 (0.70, 14.32)	515 (80, 1352)	7.15 (1.12, 18.79)	2.11 (1.59, 2.63)
Albania	15 (2, 31)	1.74 (0.27, 3.77)	16 (3, 38)	2.65 (0.41, 6.18)	1.72 (1.27, 2.19)
Algeria	173 (32, 339)	2.98 (0.56, 5.83)	600 (114, 1179)	5.35 (1.01, 10.51)	1.86 (1.80, 1.91)
American Samoa	2 (0, 4)	16.03 (3.09, 30.88)	4 (1, 8)	36.76 (6.78, 69.75)	3.02 (2.85, 3.19)
Andorra	1 (0, 2)	7.75 (1.42, 16.11)	2 (0, 4)	7.84 (1.26, 17.82)	0.55 (0.34, 0.77)
Angola	55 (10, 121)	2.41 (0.45, 5.25)	266 (45, 602)	3.47 (0.58, 7.85)	1.45 (1.28, 1.62)
Antigua and Barbuda	1 (0, 3)	7.65 (1.48, 15.75)	2 (0, 5)	9.77 (1.82, 19.33)	1.43 (1.21, 1.65)
Argentine Republic	432 (72, 952)	5.38 (0.90, 11.86)	442 (71, 926)	3.71 (0.60, 7.79)	-1.49 (-1.79, -1.19)
Armenia	38 (7, 88)	4.44 (0.79, 10.13)	16 (3, 36)	2.22 (0.36, 4.93)	-2.61 (-3.34, -1.88)
Australia	570 (117, 1103)	12.76 (2.63, 24.71)	510 (108, 924)	8.46 (1.79, 15.35)	-1.53 (-1.65, -1.41)
Austria	177 (33, 337)	8.94 (1.66, 17.05)	91 (18, 183)	4.59 (0.91, 9.28)	-2.00 (-2.17, -1.82)

Azerbaijan	50 (10, 109)	2.66 (0.51, 5.83)	74 (11, 164)	2.71 (0.39, 5.97)	-0.19 (-0.53, 0.15)
Bahamas	9 (2, 18)	12.73 (2.21, 24.99)	19 (4, 42)	18.06 (3.58, 38.55)	1.19 (0.97, 1.42)
Bahrain	9 (2, 18)	7.81 (1.35, 15.38)	38 (6, 76)	11.71 (1.93, 23.41)	1.12 (0.75, 1.49)
Bangladesh	397 (69, 863)	1.61 (0.28, 3.50)	1612 (266, 3479)	3.51 (0.58, 7.56)	3.03 (2.71, 3.35)
Barbados	13 (3, 24)	19.39 (3.74, 34.63)	17 (3, 31)	23.35 (4.45, 44.05)	0.67 (0.34, 1.00)
Belarus	107 (19, 236)	4.22 (0.74, 9.32)	83 (14, 188)	3.90 (0.66, 8.87)	-1.45 (-1.84, -1.05)
Belgium	316 (59, 624)	12.98 (2.43, 25.61)	151 (27, 298)	6.10 (1.10, 12.03)	-2.92 (-3.20, -2.63)
Belize	1 (0, 2)	2.30 (0.38, 4.62)	5 (1, 11)	4.50 (0.99, 8.99)	2.46 (2.08, 2.85)
Benin	6 (1, 15)	0.56 (0.07, 1.40)	22 (3, 55)	0.68 (0.10, 1.69)	0.24 (-0.14, 0.63)
Bermuda	2 (0, 4)	10.57 (2.25, 20.80)	1 (0, 2)	8.83 (1.80, 17.57)	-1.17 (-1.33, -1.02)
Bhutan	7 (1, 13)	4.73 (0.90, 9.36)	15 (2, 30)	7.08 (1.20, 14.54)	0.81 (0.63, 0.98)
Bolivarian Republic of Venezuela	151 (25, 297)	3.12 (0.52, 6.13)	433 (73, 970)	6.31 (1.07, 14.14)	1.87 (1.59, 2.16)
Bosnia and Herzegovina	30 (4, 65)	2.60 (0.38, 5.56)	21 (4, 48)	2.86 (0.56, 6.57)	0.44 (0.17, 0.71)
Botswana	15 (2, 33)	4.67 (0.77, 10.15)	52 (8, 114)	7.60 (1.17, 16.81)	1.96 (1.52, 2.41)
Brazil	1936 (410, 3758)	4.97 (1.05, 9.65)	4926 (954, 9681)	8.39 (1.63, 16.49)	1.54 (1.48, 1.60)
Brunei Darussalam	5 (1, 10)	7.11 (1.27, 14.74)	13 (2, 26)	10.15 (1.76, 20.58)	1.58 (1.25, 1.90)
Bulgaria	160 (25, 318)	7.71 (1.22, 15.34)	112 (20, 234)	7.87 (1.39, 16.44)	0.03 (-0.23, 0.30)
Burkina Faso	47 (8, 106)	2.27 (0.37, 5.08)	123 (20, 264)	2.24 (0.36, 4.81)	0.05 (-0.24, 0.33)
Burundi	24 (4, 53)	1.88 (0.33, 4.25)	54 (8, 126)	1.74 (0.26, 4.03)	-0.98 (-1.28, -0.69)
Cabo Verde	2 (0, 5)	2.62 (0.43, 5.91)	4 (1, 9)	2.77 (0.48, 5.93)	0.25 (0.03, 0.47)
Cambodia	25 (3, 63)	0.99 (0.12, 2.53)	77 (14, 197)	1.70 (0.30, 4.37)	1.29 (1.09, 1.50)

Cameroon	61 (9, 126)	2.55 (0.36, 5.28)	263 (41, 621)	3.36 (0.53, 7.93)	0.80 (0.64, 0.95)
Canada	293 (47, 639)	3.99 (0.63, 8.68)	220 (39, 513)	2.66 (0.47, 6.19)	-1.50 (-1.61, -1.39)
Central African Republic	20 (3, 47)	3.08 (0.51, 7.26)	53 (9, 121)	3.80 (0.62, 8.71)	0.55 (0.40, 0.69)
Chad	18 (2, 40)	1.32 (0.18, 3.02)	65 (9, 155)	1.68 (0.23, 4.00)	0.98 (0.82, 1.13)
Chile	141 (27, 289)	3.91 (0.76, 8.00)	162 (27, 340)	3.44 (0.58, 7.22)	-0.17 (-0.33, -0.01)
China	7681 (1390, 15241)	2.38 (0.43, 4.73)	10692 (1709, 23095)	3.35 (0.54, 7.25)	0.82 (0.69, 0.94)
Colombia	497 (99, 941)	5.72 (1.15, 10.84)	1254 (230, 2397)	9.57 (1.76, 18.30)	2.21 (1.75, 2.67)
Comoros	2 (0, 4)	1.74 (0.24, 4.12)	8 (1, 19)	4.01 (0.63, 9.60)	3.02 (2.52, 3.52)
Cook Islands	1 (0, 2)	17.86 (3.37, 35.34)	1 (0, 2)	20.50 (3.95, 41.77)	0.99 (0.78, 1.20)
Costa Rica	19 (3, 42)	2.48 (0.43, 5.36)	56 (10, 118)	4.31 (0.80, 9.15)	1.69 (1.50, 1.89)
Croatia	54 (11, 113)	4.50 (0.90, 9.41)	35 (5, 77)	3.94 (0.60, 8.61)	-0.49 (-0.73, -0.25)
Cuba	188 (34, 379)	6.13 (1.09, 12.35)	160 (25, 322)	6.42 (0.99, 12.96)	0.30 (0.07, 0.53)
Cyprus	13 (2, 27)	6.34 (0.94, 13.58)	19 (3, 40)	5.28 (0.79, 11.27)	-0.95 (-1.16, -0.75)
Czech Republic	128 (23, 251)	4.95 (0.88, 9.74)	102 (20, 212)	4.46 (0.86, 9.23)	-0.61 (-0.99, -0.24)
Democratic People's Republic of Korea	202 (31, 453)	3.57 (0.55, 7.99)	342 (56, 766)	5.19 (0.86, 11.62)	1.67 (1.52, 1.82)
Democratic Republic of the Congo	181 (33, 417)	2.13 (0.39, 4.88)	635 (122, 1496)	2.98 (0.57, 7.02)	1.00 (0.67, 1.34)
Denmark	160 (29, 323)	12.26 (2.20, 24.72)	55 (9, 117)	4.32 (0.72, 9.26)	-3.43 (-3.53, -3.32)
Djibouti	2 (0, 5)	2.05 (0.31, 5.21)	12 (2, 26)	3.58 (0.55, 8.24)	1.85 (1.69, 2.00)
Dominica	1 (0, 2)	5.44 (0.91, 11.77)	1 (0, 3)	8.33 (1.70, 16.62)	1.69 (1.55, 1.82)
Dominican Republic	59 (11, 120)	3.15 (0.57, 6.33)	138 (24, 297)	4.76 (0.81, 10.24)	1.63 (1.41, 1.86)
Ecuador	38 (6,	1.50 (0.25, 3.13)	113 (19,	2.40 (0.41,	1.62 (1.40,

	79)		262)	5.55)	1.83)
Egypt	820 (154, 1583)	6.25 (1.18, 12.07)	2130 (451, 4169)	8.21 (1.74, 16.07)	0.63 (0.49, 0.78)
El Salvador	23 (4, 48)	1.79 (0.30, 3.64)	60 (10, 132)	3.37 (0.56, 7.44)	2.18 (2.04, 2.31)
Equatorial Guinea	2 (0, 5)	2.44 (0.42, 5.35)	15 (2, 39)	4.19 (0.51, 10.77)	1.82 (1.69, 1.95)
Eritrea	9 (1, 23)	1.12 (0.17, 2.98)	30 (4, 80)	1.81 (0.27, 4.86)	1.71 (1.54, 1.88)
Estonia	14 (2, 30)	3.70 (0.61, 7.86)	7 (1, 16)	2.67 (0.44, 5.89)	-1.78 (-2.10, -1.46)
Eswatini	6 (1, 12)	3.06 (0.52, 6.41)	21 (4, 52)	6.71 (1.12, 16.38)	2.83 (2.41, 3.26)
Ethiopia	283 (36, 731)	2.51 (0.32, 6.48)	841 (134, 1691)	3.04 (0.48, 6.10)	0.56 (0.26, 0.86)
Federated States of Micronesia	4 (1, 9)	19.14 (3.99, 40.53)	7 (1, 15)	28.58 (5.57, 58.46)	1.29 (1.17, 1.41)
Fiji	33 (6, 66)	16.63 (3.14, 33.88)	47 (10, 100)	20.77 (4.34, 43.88)	0.95 (0.76, 1.14)
Finland	110 (20, 231)	8.75 (1.58, 18.29)	45 (7, 94)	3.96 (0.61, 8.25)	-3.06 (-3.30, -2.83)
French Republic	1442 (230, 2781)	9.97 (1.59, 19.22)	1032 (182, 2041)	7.27 (1.28, 14.38)	-1.22 (-1.49, -0.95)
Gabonese Republic	4 (1, 10)	1.94 (0.33, 4.59)	14 (2, 34)	2.89 (0.48, 7.00)	1.11 (0.92, 1.29)
Gambia	2 (0, 4)	0.77 (0.12, 1.72)	9 (2, 21)	1.47 (0.26, 3.38)	1.73 (1.41, 2.05)
Georgia	81 (15, 171)	5.94 (1.08, 12.42)	39 (6, 84)	4.97 (0.73, 10.62)	-0.52 (-0.81, -0.23)
Germany	1477 (243, 3237)	7.60 (1.25, 16.66)	768 (121, 1632)	4.51 (0.71, 9.58)	-1.72 (-1.87, -1.57)
Ghana	152 (21, 311)	4.31 (0.60, 8.82)	478 (79, 1054)	5.24 (0.87, 11.55)	0.43 (0.00, 0.87)
Greenland	1 (0, 2)	6.38 (1.00, 14.42)	1 (0, 1)	4.16 (0.61, 10.44)	-1.33 (-1.79, -0.88)
Grenada	2 (0, 3)	8.68 (1.64, 17.94)	3 (0, 6)	11.17 (1.90, 22.36)	1.06 (0.72, 1.41)
Guam	1 (0, 2)	3.25 (0.62, 6.79)	2 (0, 5)	6.01 (1.00, 12.62)	2.41 (2.03, 2.79)
Guatemala	10 (2, 24)	0.57 (0.09, 1.31)	50 (7, 111)	1.14 (0.16, 2.54)	2.35 (1.80, 2.90)
Guinea-Bissau	7 (1,	3.20 (0.47, 7.01)	23 (3,	4.36 (0.61,	0.94 (0.80,

	16)		50)	9.53)	1.08)
Guyana	12 (2, 23)	5.70 (1.00, 11.41)	21 (4, 43)	10.14 (1.85, 21.24)	2.52 (1.99, 3.06)
Haiti	106 (18, 263)	6.91 (1.16, 17.09)	317 (57, 706)	8.95 (1.62, 19.93)	0.87 (0.78, 0.96)
Hellenic Republic	154 (24, 319)	6.11 (0.96, 12.66)	114 (19, 254)	5.25 (0.86, 11.74)	-0.61 (-0.91, -0.32)
Honduras	18 (3, 40)	1.71 (0.26, 3.75)	72 (15, 172)	2.53 (0.51, 6.05)	1.05 (0.91, 1.18)
Hungary	165 (31, 346)	6.52 (1.21, 13.65)	107 (15, 218)	4.99 (0.68, 10.17)	-1.26 (-1.48, -1.05)
Iceland	5 (1, 10)	7.65 (1.29, 15.30)	4 (1, 9)	5.60 (1.07, 11.47)	-1.55 (-1.81, -1.30)
India	3918 (799, 7525)	1.94 (0.40, 3.73)	9511 (1894, 18983)	2.51 (0.50, 5.02)	0.61 (0.38, 0.84)
Indonesia	2360 (453, 5090)	4.92 (0.94, 10.62)	6299 (1098, 12976)	8.37 (1.46, 17.23)	1.84 (1.80, 1.88)
Iran (Islamic Republic of)	452 (82, 841)	3.58 (0.65, 6.65)	1783 (319, 3350)	7.67 (1.37, 14.41)	2.85 (2.37, 3.33)
Iraq	286 (55, 522)	7.04 (1.37, 12.87)	1237 (248, 2472)	11.73 (2.35, 23.45)	1.59 (1.46, 1.72)
Ireland	103 (20, 200)	11.68 (2.25, 22.61)	88 (16, 169)	7.56 (1.33, 14.46)	-1.46 (-1.62, -1.30)
Israel	109 (19, 220)	8.90 (1.56, 18.07)	107 (22, 221)	4.83 (1.00, 9.94)	-2.18 (-2.37, -1.99)
Italy	1708 (296, 3182)	11.95 (2.07, 22.26)	989 (175, 1968)	8.15 (1.44, 16.22)	-1.11 (-1.23, -0.98)
Jamaica	49 (10, 91)	8.30 (1.73, 15.25)	154 (32, 311)	20.00 (4.10, 40.29)	2.85 (2.38, 3.32)
Japan	2426 (458, 4479)	7.56 (1.43, 13.95)	2070 (387, 3995)	8.33 (1.55, 16.07)	0.18 (0.01, 0.34)
Jordan	35 (7, 74)	4.16 (0.79, 8.87)	164 (31, 333)	5.29 (1.00, 10.76)	0.82 (0.52, 1.12)
Kazakhstan	138 (26, 282)	3.36 (0.62, 6.86)	117 (19, 270)	2.47 (0.41, 5.70)	-1.09 (-1.48, -0.69)
Kenya	142 (22, 313)	2.73 (0.42, 6.04)	781 (145, 1622)	5.89 (1.09, 12.22)	2.65 (2.58, 2.72)
Kiribati	3 (1, 6)	15.61 (2.94, 30.78)	7 (1, 15)	23.37 (4.44, 47.29)	1.33 (1.19, 1.48)
Kuwait	28 (6,	6.66 (1.33, 11.89)	148 (31,	10.14 (2.10,	-0.42 (-1.26,

	49)		280)	19.13)	0.43)
Kyrgyz Republic	27 (5, 61)	2.60 (0.50, 5.79)	34 (5, 74)	1.96 (0.27, 4.31)	-1.36 (-1.60, -1.12)
Lao People's Democratic Republic	16 (2, 43)	1.65 (0.23, 4.44)	50 (8, 134)	2.51 (0.39, 6.73)	1.44 (1.25, 1.64)
Latvia	45 (9, 95)	6.88 (1.45, 14.69)	20 (4, 39)	5.06 (0.98, 9.94)	-1.46 (-1.69, -1.22)
Lebanese Republic	43 (7, 97)	5.74 (0.88, 12.98)	110 (21, 221)	7.43 (1.38, 14.90)	0.87 (0.72, 1.03)
Lesotho	3 (0, 9)	0.89 (0.12, 2.30)	9 (1, 21)	1.68 (0.20, 4.21)	2.51 (2.07, 2.95)
Liberia	19 (4, 39)	3.43 (0.65, 7.06)	94 (14, 204)	6.73 (1.03, 14.61)	1.75 (1.42, 2.09)
Libya	30 (6, 63)	3.34 (0.63, 6.89)	172 (33, 349)	8.67 (1.65, 17.55)	3.58 (3.42, 3.75)
Lithuania	48 (9, 98)	5.21 (1.03, 10.60)	28 (5, 59)	4.95 (0.85, 10.38)	-0.15 (-0.40, 0.11)
Luxembourg	9 (2, 19)	9.64 (1.82, 19.42)	7 (1, 15)	4.44 (0.89, 9.61)	-2.56 (-2.80, -2.31)
Madagascar	55 (8, 124)	2.02 (0.30, 4.62)	183 (25, 427)	2.53 (0.35, 5.89)	0.65 (0.49, 0.82)
Malawi	20 (3, 48)	0.89 (0.12, 2.13)	62 (9, 154)	1.24 (0.19, 3.07)	0.94 (0.45, 1.43)
Malaysia	271 (47, 535)	6.04 (1.04, 11.94)	898 (172, 1869)	10.66 (2.04, 22.18)	1.88 (1.65, 2.11)
Maldives	2 (0, 6)	5.24 (0.69, 13.52)	8 (2, 15)	6.60 (1.30, 12.81)	0.58 (0.45, 0.70)
Malta	13 (2, 26)	13.61 (2.58, 27.40)	10 (2, 19)	10.62 (2.08, 19.93)	-1.40 (-1.66, -1.14)
Marshall Islands	2 (0, 3)	15.71 (3.22, 32.45)	5 (1, 10)	31.48 (5.91, 68.78)	2.17 (2.01, 2.33)
Mauritania	17 (3, 34)	3.58 (0.67, 7.33)	60 (11, 121)	5.58 (0.99, 11.27)	1.33 (1.21, 1.46)
Mauritius	6 (1, 14)	2.16 (0.37, 4.53)	21 (4, 47)	6.64 (1.23, 14.89)	2.73 (2.29, 3.17)
Monaco	1 (0, 2)	13.45 (2.44, 27.37)	1 (0, 2)	14.83 (2.63, 32.05)	0.45 (0.21, 0.70)
Mongolia	3 (0, 8)	0.61 (0.09, 1.48)	8 (1, 17)	0.93 (0.18, 2.00)	1.06 (0.87, 1.25)
Montenegro	8 (2, 18)	5.39 (0.96, 11.22)	8 (1, 18)	5.75 (0.85, 12.67)	0.54 (0.13, 0.96)
Morocco	188 (33, 384)	2.98 (0.52, 6.10)	682 (116, 1470)	7.05 (1.20, 15.18)	2.95 (2.84, 3.06)

Mozambique	54 (8, 129)	1.71 (0.24, 4.07)	173 (27, 428)	2.28 (0.36, 5.63)	1.30 (1.07, 1.52)
Myanmar	162 (20, 404)	1.55 (0.19, 3.88)	311 (46, 775)	2.06 (0.31, 5.12)	0.89 (0.62, 1.16)
Namibia	12 (3, 24)	3.56 (0.76, 7.10)	51 (10, 115)	7.69 (1.46, 17.38)	2.68 (2.60, 2.76)
Nauru	0 (0, 1)	16.73 (2.75, 38.66)	1 (0, 1)	20.53 (3.83, 48.66)	0.56 (0.42, 0.71)
Nepal	52 (8, 120)	1.15 (0.17, 2.62)	140 (23, 358)	1.54 (0.25, 3.94)	1.07 (0.64, 1.50)
Netherlands	292 (52, 639)	7.37 (1.32, 16.12)	142 (26, 304)	3.87 (0.70, 8.29)	-2.43 (-2.63, -2.23)
New Zealand	81 (14, 158)	8.97 (1.56, 17.45)	91 (18, 179)	7.61 (1.51, 15.00)	0.09 (-0.11, 0.29)
Nicaragua	12 (2, 24)	1.32 (0.26, 2.71)	40 (7, 89)	2.21 (0.41, 4.89)	1.76 (1.60, 1.93)
Niger	26 (4, 61)	1.49 (0.26, 3.48)	74 (11, 173)	1.39 (0.21, 3.24)	-0.18 (-0.25, -0.10)
Nigeria	642 (92, 1300)	3.17 (0.45, 6.42)	2669 (435, 5981)	4.65 (0.76, 10.42)	1.53 (1.37, 1.69)
Niue	0 (0, 0)	14.95 (2.57, 29.83)	0 (0, 0)	20.55 (4.15, 40.68)	0.60 (0.47, 0.73)
North Macedonia	32 (5, 68)	6.28 (1.07, 13.28)	27 (5, 61)	5.16 (1.03, 11.55)	-0.94 (-1.21, -0.67)
Northern Mariana Islands	2 (0, 3)	11.64 (1.98, 24.20)	2 (0, 4)	17.53 (3.74, 33.97)	1.56 (1.10, 2.02)
Norway	78 (14, 157)	7.37 (1.32, 14.83)	46 (8, 95)	3.80 (0.67, 7.84)	-2.66 (-2.91, -2.41)
Oman	5 (1, 10)	1.40 (0.25, 3.00)	18 (3, 35)	1.76 (0.30, 3.49)	0.72 (0.57, 0.86)
Pakistan	848 (152, 1740)	3.58 (0.64, 7.36)	3435 (616, 7255)	5.67 (1.02, 11.98)	1.03 (0.86, 1.19)
Palau	1 (0, 1)	18.00 (3.58, 36.26)	1 (0, 2)	24.47 (4.51, 49.97)	0.83 (0.71, 0.94)
Palestine	40 (8, 80)	8.97 (1.75, 18.17)	142 (25, 261)	10.88 (1.90, 20.05)	0.68 (0.60, 0.76)
Panama	15 (2, 32)	2.44 (0.39, 5.18)	47 (9, 96)	4.39 (0.81, 9.01)	2.31 (2.12, 2.51)
Papua New Guinea	88 (16, 187)	9.11 (1.65, 19.37)	316 (54, 671)	12.06 (2.06, 25.66)	0.83 (0.68, 0.98)
Paraguay	17 (3, 40)	1.86 (0.29, 4.24)	52 (8, 126)	2.73 (0.45, 6.65)	1.08 (0.99, 1.17)
Peru	126 (19, 277)	2.31 (0.35, 5.10)	244 (39, 597)	2.53 (0.40, 6.21)	-0.04 (-0.26, 0.17)

Philippines	431 (74, 895)	2.77 (0.48, 5.77)	1408 (232, 3080)	4.80 (0.79, 10.51)	2.01 (1.82, 2.19)
Plurinational State of Bolivia	40 (6, 96)	2.61 (0.38, 6.30)	104 (20, 240)	3.33 (0.66, 7.71)	0.51 (0.39, 0.64)
Poland	608 (131, 1193)	6.47 (1.39, 12.70)	499 (86, 996)	5.65 (0.97, 11.28)	-1.19 (-1.50, -0.88)
Portuguese Republic	232 (41, 449)	9.18 (1.62, 17.76)	169 (25, 337)	7.27 (1.09, 14.46)	-0.63 (-0.80, -0.45)
Puerto Rico	71 (15, 141)	7.40 (1.55, 14.75)	46 (10, 94)	6.15 (1.31, 12.48)	-0.84 (-1.07, -0.62)
Qatar	10 (2, 19)	12.16 (2.27, 24.32)	55 (10, 110)	9.91 (1.81, 19.78)	-0.89 (-1.31, -0.46)
Republic of Côte d'Ivoire	50 (8, 110)	1.83 (0.30, 4.02)	208 (36, 455)	3.13 (0.54, 6.83)	2.07 (1.85, 2.30)
Republic of Guinea	20 (2, 46)	1.47 (0.18, 3.38)	62 (8, 150)	1.86 (0.25, 4.52)	0.59 (0.41, 0.77)
Republic of Korea	412 (81, 754)	3.26 (0.64, 5.97)	666 (129, 1320)	5.75 (1.11, 11.39)	1.98 (1.73, 2.23)
Republic of Mali	46 (8, 95)	2.40 (0.40, 4.97)	137 (19, 336)	2.52 (0.35, 6.18)	0.03 (-0.14, 0.20)
Republic of Moldova	51 (9, 111)	4.54 (0.76, 9.89)	32 (4, 68)	3.59 (0.50, 7.69)	-1.40 (-1.71, -1.10)
Republic of the Congo	20 (3, 52)	3.51 (0.52, 9.20)	91 (13, 213)	6.35 (0.89, 14.84)	1.94 (1.64, 2.23)
Romania	384 (75, 771)	6.84 (1.34, 13.71)	277 (51, 579)	6.81 (1.26, 14.26)	-0.12 (-0.40, 0.16)
Russian Federation	1262 (218, 2555)	3.41 (0.59, 6.90)	1195 (190, 2512)	3.53 (0.56, 7.41)	-0.75 (-1.08, -0.43)
Rwanda	44 (6, 105)	2.70 (0.34, 6.47)	95 (14, 243)	2.69 (0.38, 6.91)	-0.76 (-1.16, -0.36)
Saint Kitts and Nevis	1 (0, 2)	11.88 (2.23, 23.50)	1 (0, 3)	8.20 (1.53, 17.49)	-1.29 (-1.53, -1.04)
Saint Lucia	3 (0, 6)	8.07 (1.37, 16.46)	5 (1, 10)	11.14 (2.21, 22.29)	1.57 (1.24, 1.89)
Saint Vincent and the Grenadines	2 (0, 5)	8.48 (1.65, 17.11)	4 (1, 8)	13.89 (2.66, 30.22)	1.67 (1.47, 1.87)
Samoa	4 (1, 7)	10.10 (2.36, 19.49)	8 (2, 16)	16.02 (3.19, 32.09)	1.66 (1.52, 1.81)
San Marino	0 (0, 1)	5.26 (0.93, 10.68)	0 (0, 1)	4.62 (0.78, 11.35)	0.85 (0.41, 1.30)
Sao Tome and Principe	0 (0, 1)	1.44 (0.23, 3.18)	2 (0, 4)	2.88 (0.54, 6.52)	2.19 (2.01, 2.37)
Saudi Arabia	107 (19,	3.27 (0.59, 6.99)	857 (167,	8.45 (1.65,	3.23 (3.17,

	228)		1717)	16.92)	3.30)
Senegal	33 (5, 73)	1.94 (0.29, 4.24)	118 (20, 264)	3.04 (0.52, 6.76)	1.57 (1.37, 1.77)
Serbia	149 (24, 309)	6.40 (1.04, 13.23)	108 (19, 235)	5.31 (0.94, 11.57)	-1.17 (-1.51, -0.82)
Seychelles	1 (0, 1)	2.99 (0.48, 6.21)	2 (0, 3)	6.23 (1.06, 13.15)	3.02 (2.61, 3.42)
Sierra Leone	16 (2, 37)	1.64 (0.24, 3.69)	62 (8, 138)	2.71 (0.37, 6.05)	1.76 (1.68, 1.84)
Singapore	63 (12, 124)	6.75 (1.31, 13.25)	70 (13, 138)	4.81 (0.86, 9.46)	-1.38 (-1.63, -1.13)
Slovak Republic	65 (11, 130)	4.87 (0.82, 9.81)	50 (9, 111)	3.95 (0.69, 8.78)	-1.01 (-1.22, -0.80)
Slovenia	24 (4, 49)	4.89 (0.79, 9.84)	12 (2, 26)	2.81 (0.54, 6.20)	-1.69 (-1.98, -1.41)
Solomon Islands	5 (1, 10)	6.21 (1.07, 13.00)	25 (5, 50)	14.33 (2.78, 29.26)	3.08 (2.94, 3.22)
Somalia	29 (4, 76)	1.69 (0.26, 4.50)	77 (11, 193)	1.60 (0.24, 4.00)	-0.69 (-0.95, -0.44)
South Africa	853 (176, 1549)	8.83 (1.82, 16.04)	1748 (388, 3148)	11.29 (2.50, 20.32)	1.40 (0.95, 1.86)
South Sudan	17 (3, 42)	1.35 (0.21, 3.25)	44 (6, 106)	1.88 (0.27, 4.55)	1.18 (0.71, 1.65)
Spain	809 (139, 1648)	8.40 (1.44, 17.12)	597 (99, 1276)	6.03 (1.00, 12.89)	-0.87 (-1.03, -0.70)
Sri Lanka	69 (12, 162)	1.50 (0.26, 3.53)	130 (21, 298)	2.30 (0.37, 5.28)	2.00 (1.76, 2.25)
Sudan	252 (52, 595)	5.39 (1.11, 12.71)	988 (169, 2119)	8.75 (1.50, 18.77)	1.74 (1.53, 1.94)
Suriname	6 (1, 12)	6.38 (1.09, 12.28)	14 (3, 27)	9.45 (1.80, 18.94)	1.39 (1.23, 1.55)
Sweden	156 (28, 340)	7.62 (1.37, 16.57)	79 (17, 151)	3.58 (0.77, 6.89)	-1.92 (-2.16, -1.68)
Swiss Confederation	134 (27, 285)	7.63 (1.51, 16.21)	71 (11, 144)	3.64 (0.56, 7.37)	-2.29 (-2.63, -1.95)
Syrian Arab Republic	117 (22, 237)	4.24 (0.78, 8.59)	281 (49, 614)	7.22 (1.27, 15.78)	1.91 (1.51, 2.31)
Taiwan (Province of China)	333 (68, 649)	6.05 (1.24, 11.80)	573 (115, 1133)	10.21 (2.05, 20.18)	1.40 (1.09, 1.72)
Tajikistan	23 (4, 49)	1.89 (0.32, 4.03)	48 (6, 121)	1.90 (0.25, 4.78)	-0.28 (-0.45, -0.11)
Thailand	422 (58, 943)	2.64 (0.36, 5.90)	965 (154, 2242)	5.92 (0.94, 13.75)	2.59 (2.20, 2.97)

Timor-Leste	2 (0, 6)	1.23 (0.21, 3.03)	6 (1, 15)	1.86 (0.29, 4.33)	1.03 (0.66, 1.41)
Togolese Republic	9 (1, 21)	1.07 (0.17, 2.41)	35 (5, 82)	1.60 (0.21, 3.81)	1.19 (0.87, 1.50)
Tokelau	0 (0, 0)	14.74 (2.45, 30.91)	0 (0, 0)	20.99 (4.13, 41.62)	0.80 (0.66, 0.95)
Tonga	4 (1, 7)	17.05 (3.38, 32.04)	5 (1, 11)	21.13 (4.28, 43.38)	0.72 (0.66, 0.77)
Trinidad and Tobago	38 (7, 73)	12.32 (2.38, 23.75)	66 (12, 135)	19.55 (3.65, 39.76)	1.11 (0.91, 1.30)
Tunisia	31 (5, 66)	1.52 (0.25, 3.23)	101 (17, 212)	3.28 (0.55, 6.90)	2.54 (2.47, 2.60)
Turkey	224 (41, 466)	1.57 (0.29, 3.27)	1294 (240, 2627)	5.97 (1.11, 12.13)	6.29 (5.32, 7.27)
Turkmenistan	17 (3, 35)	1.92 (0.33, 4.00)	38 (6, 90)	3.01 (0.47, 7.18)	1.83 (1.44, 2.22)
Tuvalu	0 (0, 1)	13.88 (2.54, 30.11)	0 (0, 1)	15.62 (3.02, 32.88)	0.11 (-0.10, 0.33)
Uganda	57 (9, 134)	1.48 (0.22, 3.48)	274 (41, 666)	2.63 (0.40, 6.40)	1.03 (0.69, 1.36)
Ukraine	635 (101, 1409)	5.02 (0.80, 11.13)	348 (57, 806)	3.44 (0.57, 7.97)	-2.06 (-2.40, -1.71)
United Arab Emirates	17 (3, 36)	4.98 (0.99, 10.43)	147 (26, 303)	8.50 (1.53, 17.58)	1.32 (0.91, 1.72)
United Kingdom	2915 (576, 5243)	20.54 (4.06, 36.94)	1636 (296, 2995)	10.66 (1.93, 19.52)	-2.25 (-2.36, -2.14)
United Mexican States	509 (96, 980)	2.33 (0.44, 4.48)	1415 (267, 2893)	4.04 (0.76, 8.25)	1.63 (1.34, 1.92)
United Republic of Tanzania	47 (5, 120)	0.78 (0.09, 2.00)	146 (22, 358)	0.98 (0.15, 2.41)	0.38 (0.18, 0.59)
United States of America	4783 (1011, 9428)	7.14 (1.51, 14.07)	3656 (682, 7266)	4.83 (0.90, 9.60)	-1.32 (-1.46, -1.18)
United States Virgin Islands	3 (0, 5)	8.87 (1.27, 18.80)	1 (0, 3)	8.46 (1.16, 19.77)	0.74 (0.41, 1.07)
Uruguay	66 (11, 140)	8.73 (1.50, 18.59)	70 (12, 146)	8.42 (1.48, 17.51)	-0.17 (-0.32, -0.01)
Uzbekistan	66 (9, 139)	1.34 (0.18, 2.84)	197 (37, 405)	2.21 (0.42, 4.55)	1.78 (1.60, 1.95)
Vanuatu	0 (0, 1)	1.30 (0.16, 3.28)	2 (0, 4)	2.03 (0.33, 4.97)	0.65 (0.38, 0.92)
Viet Nam	267 (37, 654)	1.57 (0.22, 3.83)	986 (172, 2117)	3.86 (0.68, 8.29)	3.27 (3.17, 3.36)

Yemen	54 (10, 113)	1.99 (0.36, 4.13)	332 (64, 759)	3.98 (0.76, 9.08)	2.29 (2.10, 2.49)
Zambia	45 (7, 107)	2.43 (0.40, 5.81)	252 (31, 666)	5.13 (0.62, 13.54)	2.68 (2.09, 3.28)
Zimbabwe	41 (8, 88)	1.71 (0.34, 3.68)	191 (27, 430)	4.70 (0.66, 10.60)	4.31 (3.57, 5.05)

EAPC, estimated annual percentage change; CI, confidence interval; UI, uncertainty interval; SDI, socio-demographic index.

Supplementary Table 3

Location	Overall difference	Aging	Population growth	Epidemiological change	Aging (%)	Population growth (%)	Risk (%)
Global	1420.49	157.44	1643.14	-380.09	11.08	115.67	-26.76
Low SDI	362.25	21.11	246.38	94.76	5.83	68.01	26.16
Low-middle SDI	786.32	29.33	457.51	299.48	3.73	58.18	38.09
Middle SDI	694.41	10.37	527.13	156.91	1.49	75.91	22.60
High-middle SDI	-92.96	-9.76	159.77	-242.97	10.50	-171.87	261.37
High SDI	-283.86	-15.58	209.89	-478.17	5.49	-73.94	168.45

SDI: Socio-demographic Index

Supplementary Table 4

Location	Overall difference	Aging	Population growth	Epidemiological change	Aging (%)	Population growth (%)	Risk (%)
Global	75346.4	7305.033	82616.074	-14574.703	9.7	109.65	-19.34
Low SDI	17946.71	961.088	12122.669	4862.955	5.36	67.55	27.1
Low-middle SDI	39040.59	1174.043	22425.119	15441.428	3.01	57.44	39.55
Middle SDI	36274.6	478.257	26193.513	9602.832	1.32	72.21	26.47
High-middle SDI	-3001.72	-506.015	7950.473	-10446.176	16.86	-264.86	348.01
High SDI	-12018.72	-759.521	10722.682	-21981.883	6.32	-89.22	182.9

SDI: Socio-demographic Index
